# Physical Activity through Sustainable Transport Approaches (PASTA): protocol for a multi-centre, longitudinal study

**DOI:** 10.1186/s12889-015-2453-3

**Published:** 2015-11-14

**Authors:** Evi Dons, Thomas Götschi, Mark Nieuwenhuijsen, Audrey de Nazelle, Esther Anaya, Ione Avila-Palencia, Christian Brand, Tom Cole-Hunter, Mailin Gaupp-Berghausen, Sonja Kahlmeier, Michelle Laeremans, Natalie Mueller, Juan Pablo Orjuela, Elisabeth Raser, David Rojas-Rueda, Arnout Standaert, Erik Stigell, Tina Uhlmann, Regine Gerike, Luc Int Panis

**Affiliations:** Flemish Institute for Technological Research (VITO), Boeretang 200, 2400 Mol, Belgium; Centre for Environmental Sciences, Hasselt University, Agoralaan building D, 3590 Diepenbeek, Belgium; Physical Activity and Health Unit, Epidemiology, Biostatistics and Prevention Institute, University of Zurich, Seilergraben 49, 8001 Zurich, Switzerland; Centre for Research in Environmental Epidemiology (CREAL), C/Dr. Aiguader 88, 08003 Barcelona, Spain; Universitat Pompeu Fabra (UPF), C/Dr. Aiguader 88, 08003 Barcelona, Spain; CIBER Epidemiología y Salud Pública (CIBERESP), C/Monforte de Lemos 3-5, 28029 Madrid, Spain; Centre for Environmental Policy, Imperial College London, Exhibition Road, South Kensington Campus, SW7 2AZ London, UK; University of Oxford (UOXF) – Transport Studies Unit, South Parks Road, Oxford, OX1 3QY UK; University of Natural Resources and Life Sciences Vienna, Institute for Transport Studies, Peter-Jordan-Straße 82, 1190 Vienna, Austria; Transportation Research Institute (IMOB), Hasselt University, Wetenschapspark 5/6, 3590 Diepenbeek, Belgium; Trivector Traffic AB, Stockholm, Sweden; Dresden University of Technology, Chair of Integrated Transport Planning and Traffic Engineering, 01062 Dresden, Germany

**Keywords:** Physical activity, Walking, Cycling, Travel behaviour, Air pollution, Traffic safety, Study protocol, Longitudinal

## Abstract

**Background:**

Physical inactivity is one of the leading risk factors for non-communicable diseases, yet many are not sufficiently active. The Physical Activity through Sustainable Transport Approaches (PASTA) study aims to better understand active mobility (walking and cycling for transport solely or in combination with public transport) as an innovative approach to integrate physical activity into individuals’ everyday lives. The PASTA study will collect data of multiple cities in a longitudinal cohort design to study correlates of active mobility, its effect on overall physical activity, crash risk and exposure to traffic-related air pollution.

**Methods/Design:**

A set of online questionnaires incorporating gold standard approaches from the physical activity and transport fields have been developed, piloted and are now being deployed in a longitudinal study in seven European cities (Antwerp, Barcelona, London, Oerebro, Rome, Vienna, Zurich). In total, 14000 adults are being recruited (2000 in each city). A first questionnaire collects baseline information; follow-up questionnaires sent every 13 days collect prospective data on travel behaviour, levels of physical activity and traffic safety incidents. Self-reported data will be validated with objective data in subsamples using conventional and novel methods. Accelerometers, GPS and tracking apps record routes and activity. Air pollution and physical activity are measured to study their combined effects on health biomarkers. Exposure-adjusted crash risks will be calculated for active modes, and crash location audits are performed to study the role of the built environment. Ethics committees in all seven cities have given independent approval for the study.

**Discussion:**

The PASTA study collects a wealth of subjective and objective data on active mobility and physical activity. This will allow the investigation of numerous correlates of active mobility and physical activity using a data set that advances previous efforts in its richness, geographical coverage and comprehensiveness. Results will inform new health impact assessment models and support efforts to promote and facilitate active mobility in cities.

**Electronic supplementary material:**

The online version of this article (doi:10.1186/s12889-015-2453-3) contains supplementary material, which is available to authorized users.

## Background

Physical inactivity has emerged as a leading risk factor for non-communicable diseases, and is estimated to cause nearly 3.2 million premature deaths per year worldwide [1]. It is recommended that in a typical week adults perform at least 150 min of moderate intensity aerobic physical activity (PA), or alternatively at least 75 min of vigorous intensity aerobic PA or an equivalent combination of moderate- and vigorous-intensity activity [2]. However, globally 23 % of adults, and more than 40 % of adults in high income countries remain “physically inactive” [3], i.e. have an activity level insufficient to meet these recommendations.

Active mobility (AM), namely walking and cycling for transport solely or in combination with public transport, is well suited for integrating health-promoting PA as part of daily routines such as to travel to work or school. In contrast to sports or exercise, AM can be convenient in that it serves the dual purpose of being a mode of transport and PA, is economically affordable for most people, and provides an equitable and accessible form of PA. As such, it has the potential to reach population groups that are unresponsive to appeals and benefits of leisure time PA. In addition to the direct health benefits of PA, AM is linked to higher levels of mental wellbeing [4]; and an increase in AM may lead to city-wide improvements in air quality and additional population health benefits.

AM, however, is associated not only with health benefits, but also with certain health risks. The higher minute ventilation during PA increases the inhaled dose of traffic-related air pollutants (TRAP) [5–7]. Health risks associated with elevated exposure to air pollution are demonstrated, but evidence is mainly from long term studies estimating air pollution exposure on the home address. Short term health effects from exposure to TRAP are usually studied in a small number of people in one place in a crossover design using scripted routes and repeated measures [8, 9]. There is some evidence on the interaction between PA, air pollution exposure and health outcomes, but the evidence is incomplete and more studies are necessary [10, 11]. AM users may also be exposed to higher risks of traffic safety incidents; however, due to the potential “safety-in-numbers effect” it remains largely unclear how this risk changes when the AM shares increase [12, 13]. When estimating crash risk, not only the number of crashes, but also the exposure needs to be known preferably on a disaggregated personal level from a longitudinal study. To date, the only alternative to derive risk estimates is based on travel surveys combined with crash and injury statistics (from other subjects) which are incomplete and also not suited for any further analysis of correlates [14]. Further, the risks, costs and behavioural impact of minor crashes (collisions or falls) or near misses (an unexpected event while walking or cycling which forces someone to take sudden evasive action, without which a collision would have occurred) using AM are largely unknown [15–17].

Recent reviews conclude that on average the estimated health benefits of walking and cycling are substantially larger than associated detrimental effects of air pollution exposure and traffic safety incidents [13, 18–20]. But much uncertainty remains on several of the crucial factors.

When gathering data on issues related to PA, AM, and crashes an important limitation is the typical cross-sectional design of travel and PA surveys. Therefore they do not allow the analysis of mobility behaviour of a cohort over a longer time period or identify causal influence [21, 22]. In contrast, numerous research questions – for instance, how do AM measures influence the behaviour of inhabitants over time?; does PA from AM substitute for PA from other domains?; or what are the main user risks for crashes? - require repeated measures in a multi-period design where comparable data are gathered at regular or irregular intervals. Repeated measures of AM and PA are also warranted to derive robust estimates of long term average behaviour since both AM and PA show substantial temporal variability.

*P*hysical *A*ctivity through *S*ustainable *T*ransport *A*pproaches (PASTA) is a collaborative research project that aims to better understand AM. The PASTA project conducts a longitudinal study to look into correlates of AM and their interrelation with overall PA, crash risk and exposure to air pollution. In addition, ongoing AM initiatives are evaluated in seven European case study cities. More specifically, the study objectives are:

- ▪To evaluate the effectiveness of selected interventions and measures to promote AM with regards to increasing AM and PA;
- ▪To investigate important correlates and interrelations of AM, PA, air pollution and crash risk; and,
- ▪To fill key gaps in the understanding of AM to improve the quantitative health impact assessment (HIA) approach.

To achieve these objectives, the project uses innovative research methods including a longitudinal assessment of travel and PA behaviour, electronic and web-based data collection, exposure-adjusted assessment of crash risk from AM, innovative sensors for travel behaviour, PA and crash locations, and improved knowledge of acute health effects of AM and exposure to air pollution. All this is done in a controlled and standardised way while at the same time, putting it in the context of each case study city.

## Methods/Design

**Fig. 1:**
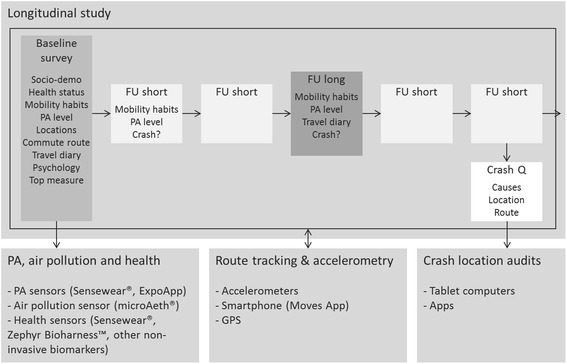
Modules and tools of the PASTA study. The core module will be implemented in all seven cities on a web-based data collection platform (goal: 2000 respondents per city), whereas the add-on modules (PA, air pollution and health; Route tracking and accelerometry; Crash location audits) will take place in selected cities. These modules aim for 120 or more participants each. FU: Follow-up questionnaire; Crash Q: Crash questionnaire; PA: Physical activity; GPS: Global Positioning System

**Fig. 2:**
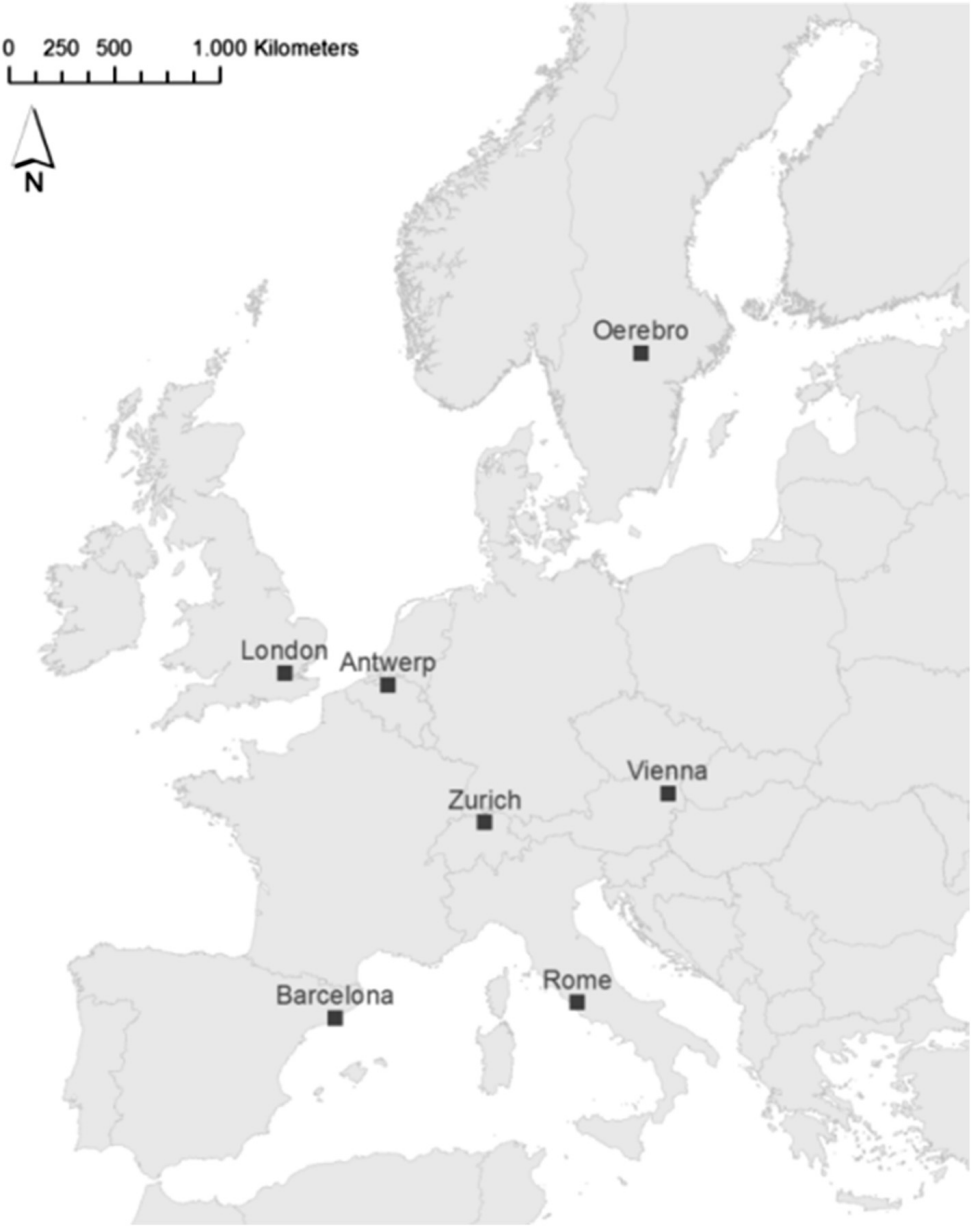
Seven cities participating in the PASTA longitudinal study

The PASTA project combines a longitudinal web-based survey with several smaller studies to gather objective data to complement the self-reported survey data (Fig. 1). An innovative web-based survey design deploys frequent follow-up questionnaires through a fully-automated platform. The survey is conducted in seven European cities: Antwerp BE, Barcelona ES, London GB, Oerebro SE, Rome IT, Vienna AT, Zurich CH (Fig. 2). Cutting edge technologies are used to collect objective (i.e. not self-reported) and relatively unbiased data in subsamples pulled from the survey respondents in some cities.

### Longitudinal online survey

#### Survey contents

**Fig. 3:**
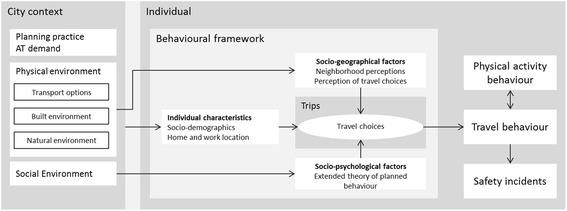
Conceptual framework

To align the survey contents with the research objectives, a detailed conceptual framework was developed based on previous work by others [23–25] and a scoping review of determinants of active mobility behaviour. Figure 3 illustrates the main domains of the framework. The framework distinguishes hierarchical levels for the various factors (i.e. city, individual, and trips), and three main domains or pathways that influence AM (and PA) behaviour, namely socio-geographical factors, socio-psychological factors, and rationale or mode choice related factors [23].

Data on contextual factors will be collected from publicly available GIS data and other data sources (i.e. weather data, population statistics, etc.) and by means of stakeholder interviews. Individual level data are being collected through the PASTA survey. Socio-psychological factors include concepts from extended theory of planned behaviour [24, 26], transtheoretical model [27] and a range of attitudinal questions. Various socio-geographical factors collected as objective city wide data are matched with corresponding questions in the survey to capture subjective perception of these same aspects (e.g. proximity of public transport). The main outcomes of interest in PASTA are travel behaviour (measured by frequency scale and 1-day travel diary adapted from KONTIV^©^ design; and commuting route identification), physical activity behaviour (measured by PA single item [28] and the Global Physical Activity Questionnaire GPAQ with walking and cycling separated [29]), and traffic safety incidents (i.e. crashes and near misses – as used for example in the SHAPES project [16]). These are collected prospectively and measured repeatedly (see survey design and Fig. 1).

#### Survey design

To address several of the research questions, PASTA uses a longitudinal design, with a comprehensive baseline questionnaire and frequent short follow-ups.

The initial baseline questionnaire takes approximately 30 min and collects key socio-demographic, individual, household, health, attitudinal and other variables that identify the person and puts her/him into social context. Frequency of use of different modes and GPAQ questions gather information on mobility and PA habits. A one-day travel diary captures trips of the previous day in much detail. Thirteen days after completion of the last questionnaire, a short follow-up questionnaire which takes only about 5 min to complete is sent to the participant asking about PA and travel behaviour in the last seven days, and crashes and near misses since the last questionnaire (Fig. 1). Each third questionnaire is a somewhat longer follow-up including also a one-day travel diary, and taking about 10 min to complete. If a crash using AM is reported in one of the follow-ups, this prompts an additional crash questionnaire asking about crash circumstances, location, causes, injuries and other consequences.

Longitudinal designs are generally considered more challenging with respect to costs and complexity, and they put a higher burden on respondents. Several measures have been put in place to reduce attrition rates and ensure high data quality. The user-friendly and custom-made survey platform gives an intuitive overview of completed and open questionnaires. If a participant has not completed or finished a questionnaire, the platform is programmed to send them e-mail reminders after the 3^rd^, 10^th^ and 20^th^ day. The participant can log-in to the platform at any time and complete the unfinished questionnaire.

Preliminary data suggest drop-out rates of 20-25 % for the first short and for the long follow-up questionnaires; and approximately 15 % for the following short follow-up questionnaires.

#### Survey sample and recruitment

Based on a power-analysis, a pooled sample of 14000 respondents - 2000 in each of the seven partner cities - will be suited to address the key questions on AM and PA as well as to conduct in depth analyses of specific correlates and subgroups. Initial experiences and emerging issues are helping us in balancing the number of people recruited with the objectives of each analysis (and the number of correlates to be modelled). A number of exclusion criteria were applied: a minimum age of 16 years (18 years depending on the local ethical approval), and to be living and/or working/studying and/or regularly travelling in a PASTA city. On enrolment, participants register on the PASTA website and give informed consent (Additional file 1: Figure S1).

A standardized recruitment strategy was developed for all cities using an opportunistic approach. This included a press release after launching the survey platform; common promotional materials including postcards and leaflets; direct targeting of local stakeholders and community groups; and extensive use of social media. Within this framework there was room for local initiatives and targeted, city-specific recruitment. A coordinated effort was set-up at the beginning of the data collection and maintained throughout as we expect recruitment to continue for the duration of the study. To minimise attrition a user engagement strategy was developed, including incentivizing participation (lottery, except in Sweden where this is not allowed), regular contact with the respondents, branding of PASTA, posting on social media, and keeping the PASTA website up-to-date.

#### Technical implementation

The survey in the core module is implemented as an online web application, with a responsive design approach (i.e., the questionnaire can be completed across a wide range of devices – from mobile phones and tablets to desktop computers).

The PASTA platform is implemented in PHP with a PostgreSQL back-end database. In addition to the participant’s user interface, it also provides a researchers’ user interface and dashboard for real-time monitoring of recruitment and survey data collection, and a survey administration interface for survey creation and management (Additional file 1: Figures S1-4). All content was developed in English and translated into Swedish, Dutch, Catalan, Spanish, Italian, Swiss German, and Austrian German using the collaborative Pootle translation tool (http://pootle.translatehouse.org/). A formal testing protocol ensured systematic testing by the project partners before the official launch of the survey platform in November 2014. The survey can be accessed here: https://survey.pastaproject.eu/ (Additional file 2) and is planned to be online until October 2016.

### Effectiveness of measures to promote active mobility

A key objective of the study is to evaluate the effectiveness of measures to promote AM with regards to their impacts on AM and PA. Other studies such as the iConnect study [25] in the UK have shown the effects of new physical infrastructures on AM and PA, using a similar approach. In comparison, the PASTA study investigates a wider range of AM measures defined here as actions or projects undertaken to increase the level of AM (in a specified population). For each city, the PASTA team is investigating one high-priority measure (hereafter ‘top measure’) to promote AM and PA (Additional file 1: Table S1). These measures range from infrastructure investments and built environment changes, such as bicycle racks and a dedicated cycling bridge, to soft measures such as workplace mobility management and individual marketing campaigns including ICT-elements.

**Fig. 4:**
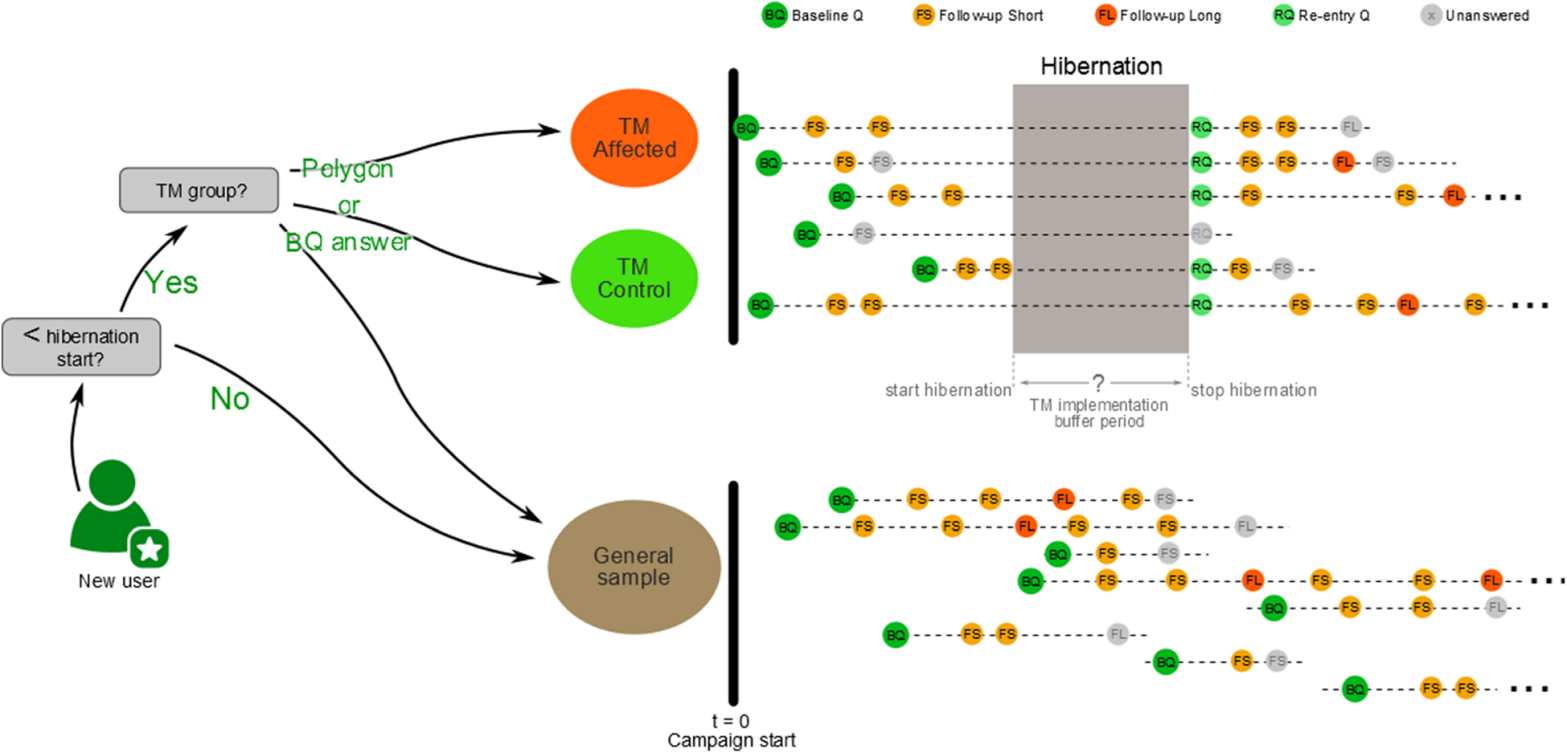
Framework of the PASTA longitudinal study design – the figure shows the questionnaire flow for the general sample and for the top measure (TM) affected and control groups. Top measure groups are selected either through a spatial query (polygon) or by a specific response in the baseline questionnaire (BQ)

Participants taking part in the longitudinal survey are identified as either being affected by the local top measure or being part of the ‘control group’, as defined by the distance between their home/work and the intervention address or by their response to a baseline questionnaire item asking about awareness and use of the relevant scheme (Fig. 4). These respondents are asked to complete a sequence of questionnaires ‘before’ (baseline and two short follow-up questionnaires) and ‘after’ the implementation of the top measure. After the implementation a re-entry and several follow-up questionnaires are sent to account for response lags (people do not change their behaviour immediately after an AM intervention). During the implementation period – referred to as the hibernation period – they do not receive any new questionnaires. A hibernation period was defined to avoid an effect of the implementation of the intervention itself (e.g. construction works) on the responses and to keep attrition rates as low as possible. For top measures that are implemented stepwise or have no clear time schedule, no hibernation phase was defined and respondents continue to receive follow-up questionnaires. Top measures will be evaluated over time (within subjects) and across study groups (i.e. affected versus control group). Power calculation indicated significant gains in power from repeated measurements before and after the implementation of the top measures, which are assumed to outweigh loss in respondents due to increased burden from repeated measurements.

### Real-life study on route tracking & accelerometry

Given the known limitations of self-assessed levels of walking and cycling [30, 31], it is important to validate self-reported (subjective) data from the PASTA longitudinal online study. In a sample of volunteers selected from the longitudinal study (about 20 % of all respondents), the smart phone application Moves will be used to track journeys and automatically detect active travel modes over the study period (https://www.moves-app.com/), as shown previously by others [32, 33]. Using the participant’s PASTA identifier, data is downloaded from the Moves server and uploaded to the PASTA server (Additional file 1: Figure S5). Objective data on walking and cycling trips can again be compared to single questionnaire items like the mode frequency scale and the travel diary, or to physical activity summary variables like the GPAQ by computing Spearman correlation coefficients [31]. One goal is to derive correction factors for self-reported versus objective travel, which will be applied in the development of a HIA. Routes data from the Moves application will be used to analyse mode and route choice, and spatial aspects of AM.

In addition, in selected cities these same participants are also invited to wear an accelerometer during one week, providing objective data on overall PA. This will further allow validation of the adapted GPAQ questionnaire included in the PASTA survey by comparing the survey-reported values with accelerometer-derived levels of walking and cycling. We know from previous studies that time spent cycling may contribute to the disagreement between self-reported and objectively measured estimates of activity [31]. Therefore new accelerometry methods (triaxial, wrist-worn) are compared to standard accelerometry, especially to assess cycling which traditionally has been hard to monitor.

Because of the low user burden, no financial compensation is rewarded.

### Real-life study on PA, air pollution & short-term health effects

We aim to collect objective data on possible health outcomes of AM. Air pollution exposure and the increased minute ventilation and dose during PA, is one of these potential negative effects. On the other hand, PA has a positive impact on health, maybe even on the same biomarkers. The study is designed as a repeated-measures study in a free-living population. In contrast to scripted studies, volunteers are tracked over a longer time period (one week) widening the window with prior information on air pollution exposure, PA levels, travel behaviour, and other confounders. Given this study design, mobile measurements of PA and exposure to TRAP are necessary; a number of previous studies managed to use new mobile sensors for this [34, 35]. By measuring air pollution and PA at the same time, the combined effects on health outcomes can be studied.

In three cities (Antwerp, Barcelona, London) a total of 120 participants are selected from the PASTA core study and equipped with tracking, air pollution and health sensors. Every participant carries all devices for one week while pursuing their regular activities (Additional file 1: Figure S6): a microAeth black carbon aerosol monitor (AethLabs, USA), a SenseWear (BodyMedia, USA) for PA, a Zephyr BioHarness (Zephyr, USA) for breathing rate and heart rate, a GPS (I-GOTU GT-600) and a smartphone (Samsung Galaxy SII, Korea) for geo-localization and accelerometry. This continuous 7-day measurement cycle is repeated in three contrasting seasons by every volunteer. At the beginning and at the end of the measurement week, volunteers visit the study centre and specific subclinical health biomarkers are measured in a controlled setting – these health markers can be linked to PA and TRAP exposure the hours and days before the measurement. The biomarkers were selected after a literature review, with non-invasive and direct read-out markers given preference. The final selection included both cardiovascular and respiratory parameters. Heart rate variability (HRV) is measured by the Zephyr BioHarness, and particle exposures have often been associated with lower HRV [8, 36–38], while blood pressure (measured by the Omron M10-IT, the Netherlands) is suspected to increase with increasing exposures to TRAP [38–40]. The microcirculation can be explored noninvasively by fundus images (Canon CR-2 Retinal Camera, Japan); the diameter of the vessels in the retina tends to decrease with an increase in TRAP [40, 41]. Exhaled NO is measured with the NIOX VERO (Aerocrine, Sweden); as a marker for lung inflammation exhaled NO generally increases after traveling and being exposed to TRAP [8, 9, 11, 36, 38, 42, 43]. Finally, a spirometry test is performed measuring lung function (EasyOne, ndd Medizintechnik AG, Switzerland). Multiple studies find no acute effects of exposure to TRAP or PA in healthy adults, others find small effects on some of the parameters [8, 9, 11, 36, 38, 43, 44]. Participants receive a small financial compensation for participation.

### Prospective study on traffic safety incidents and investigation of crash locations

Objective and perceived safety are important barriers to AM [13, 17]. In the core longitudinal survey as well as in one of the add-on modules, traffic safety incidents are studied aiming to fill some of the research gaps identified. First of all, risks of having a traffic safety incident are poorly estimated up to now. Since travel behaviour and traffic safety incidents (crashes and near misses) are tracked over time, we can calculate exposure-adjusted (per km) risk estimates and further analyse these for relevant correlates, by incident categories, and by travel mode (walking, cycling, e-bikes). Such analyses are crucial for traffic safety improvement measures. Also, minor crashes and near misses are substantially underreported [45]. By specifically asking to report minor crashes and near misses in a prospective design (thus asking regularly), reporting levels should go up, and incidents not recorded in hospital, insurance or police records will be captured.

The role of built environment attributes, in particular infrastructure, is also poorly understood in this context. PASTA will conduct infrastructure audits of crash locations reported by pedestrians and cyclists, and compare these to randomly selected non-crash locations from respondents’ routes. Only a meticulous, exposure weighted case-crossover analysis can provide risk estimates for specific infrastructure types and attributes, as previously shown by Teschke et al. [46] and Vandenbulcke et al. [47].

### Data analysis plan

The PASTA data analysis plan involves coordinated procedures for planning, organizing and documenting research, cleaning and preparing data, and analysing data. GitHub will be used as a practical tool for exchange of scripts (STATA or R), version control, issues and milestones between researchers (https://github.com/).

The collected data are automatically saved in a relational database management system (RDBMS) managed in one place. Data is anonymised before further analysis by stripping out or coding all information that would allow identification. The primary survey data is complemented with secondary data, such as GIS, meteorology, traffic, and land-use. A collection of indicators on AM measures, infrastructure, policies and relevant framework conditions is available for each city within PASTA, and can be used for qualitative data analyses.

Planned analyses cover a broad range of research questions, including building a predictor model for AM and on correlates of AM, PA and crash risk. The interrelation of AM and overall PA (‘substitution effects’) will be studied as one of the key outcomes. Qualitative data from interviews and workshops with stakeholders undertaken in parallel work in PASTA will be analysed and considered in the interpretation of findings and to the extent that it will be possible to build psychosocial constructs and latent variables, integrated in the statistical analysis and modelling. Separate analyses will focus on previously understudied topics such as e-bikes, bike-sharing and car-sharing programs. Pooled analyses comprising data from all seven cities will be preferred over single-city evaluations.

A specific objective of the PASTA project is to build a HIA on AM [19, 48], and to update the WHO Health Economic Assessment Tool (HEAT http://www.heatwalkingcycling.org/). This will help urban planners, transport planners and policy makers in the decision making process for investments in AM measures. Quantitative data from the longitudinal online survey and add-on experimental studies will provide HIA with an indication of a.o.:

- ▪The level of walking and cycling (average and distribution);
- ▪The contribution of walking and cycling to total PA;
- ▪Substitution of leisure time PA by AM;
- ▪The crash risk per kilometre walked/cycled in each city;
- ▪Air pollution exposure in different modes and its potential effects on health, and the combined effects of PA and air pollution on health.

## Discussion

The PASTA study investigates correlates and the health impact of AM, thereby addressing several limitations of previous studies (Table 1).

**Table 1:** Limitations of current work on AM and PA and how PASTA will address these (adapted from Gerike R, et al: Physical Activity Through Sustainable Transport Approaches (PASTA): A study protocol for a multi-centre project, forthcoming)

Gaps in knowledge – current state-of-the-art; limitations of current work	The PASTA approach – what we add
Few multi-centre studies in Europe with comparable research designs.	One study design is applied in seven PASTA cities (Antwerp, Barcelona, London, Oerebro, Rome, Vienna, Zurich).
Most studies on correlates of AM are cross-sectional.	Longitudinal approach, online survey with long baseline questionnaire and frequent short follow-ups, continuous recruitment over two years.
Often small sample sizes.	Targeted sample size of 14000 respondents across seven cities, number of submitted questionnaires per city > 5000.
Current studies are conducted either with methods from public health (over-simplified picture of travel behaviour, no motorised trips) or from transport research (no leisure time PA, proportion of recreational PA in leisure trips unclear).	PASTA takes an interdisciplinary approach with a systematic combination of methods from public health (modified GPAQ) and transport research (travel diary) for comprehensive data collection on AM and PA.
The relative importance of various correlates of individual AM behaviour is poorly understood, few studies comprehensively assess the wide range of factors which affect AM and PA.	Data collection and analysis based on a broad conceptual framework reflecting geographical, utilitarian and psychological factors, as well as data hierarchies (aggregation levels).
Contextual factors are usually not taken into account in quantitative analyses.	Systematic combination of qualitative and quantitative methods, with a major longitudinal web-based survey, expert interviews, stakeholder workshops, compilation of city indicators on AM, PA, and contextual factors. Qualitative data is integrated in quantitative analyses.
Substitution behaviour is poorly understood.	Multiple, repeated parallel assessments of AM and PA allow for quantification of substitution behaviour in the short and longer term. We will advance the field by also using real tracked data [50].
Few studies exist on the evaluation of AM measures.	Evaluation of top measures in the PASTA cities - infrastructure investments and built environment changes, such as bicycle racks and a dedicated cycling bridge; soft measures such as workplace mobility management and individual marketing.
Self-reported estimates of PA and AM are often not validated.	Validation of self-reported data on levels of PA and AM on subsamples collecting objective data using accelerometers, smartphone tracking apps, GPS loggers.
Lack of real-life studies on combined health effects of air pollution and PA – especially multi-centre studies are missing.	In three cities, exposure to air pollution and PA is assessed under real-life conditions. A multitude of non-invasive health biomarkers are repeatedly measured in 120 volunteers.
Air pollution exposure while traveling is largely unknown or ignored by using fixed monitoring stations.	Mobile sensors are used for air pollution, PA and travel behaviour. Not only exposure, but also inhaled dose is taken into account (especially relevant for AM).
Underreporting of minor AM crashes and near misses.	Integration of questions about AM crashes and near misses into the core module of the PASTA longitudinal survey.
Crash risk for walking and cycling is based on cross-sectional counts of fatal/reported accidents.	Exposure-adjusted crash risk (including near misses) using a longitudinal study design.

PASTA is set up as a multi-centre study. The longitudinal survey is conducted in seven European cities benefiting from the power of pooled analyses and coverage of a broad range of social backgrounds, cultures, population densities, urban form, policy measures, climates, and framework conditions. The add-on modules focus on specific research questions in a limited number of cities while collecting objective quantitative data.

The core module is designed as a longitudinal survey. The survey content is based on a newly developed, comprehensive conceptual framework. The framework combines three domains of travel demand modelling, namely a rationalist mode choice approach, a socio-geographical approach including various spatial factors, and a socio-psychological approach, building on established behavioural theories [23, 26, 49]. The baseline questionnaire is designed in a way that even respondents who may choose to drop out of the study after the baseline would provide a substantial data set suited for various cross-sectional analyses of interest. The presumed gain from front-loading the user burden is also that the burden of the fairly frequent follow-ups can be kept to a minimum and as such keep those respondents who decide to participate in the follow-up, in the study for as long as possible. Each third follow-up is somewhat longer as it also includes a one-day travel diary and the GPAQ questions with walking and cycling separated, to collect more detailed data on AM and PA at least once every season. The data analysis is designed to make use of all collected data, independent of how long respondents stay in the study.

We believe that a longitudinal online survey with rolling recruitment is innovative and essential to reach sufficient numbers of respondents within a reasonable budget. However, several limitations need to be acknowledged. Offering only the electronic platform for participation may exclude a number of specific demographics (e.g. elderly populations) and other groups (e.g. people without access to the internet). For some analyses this may lead to results that cannot be extrapolated to the wider population. Taking into account the current equality agenda, partner cities have taken initiatives specifically aimed at including local population groups that are currently at risk of being undersampled. By using this stratified and opportunistic recruitment approach informed by ongoing data monitoring, recruitment can be directed at underrepresented groups to obtain a sample that more closely reflects the characteristics of the general population. However, opportunistic recruitment is difficult to randomize, and having a biased sample is likely to be unavoidable because of the required input that is expected and self-selection. In the analyses we aim to address the bias, where needed, with survey weighing techniques.

The number of completed follow-up questionnaires gradually decreases over time as respondents drop out for various reasons. To guarantee internal validity, multi-level models will be estimated which consider the hierarchical data structure (points in time < individuals < (sub-)populations/cities). For example, for a continuous outcome variable (e.g. minutes of AT), for each respondent the first level of the model estimates an intercept and a slope. The intercept reflects the individual’s average level of AM, the slope reflects the change over time. In a second stage of the model, these individual estimates are aggregated to population average estimates. These estimates will be independent of when the individual assessments occurred and how often (i.e. adjusted for covariates on temporal factors like date of assessment (weekday, season), weather, etc.). For top measure affected and control groups, a hibernation period is built in to keep attrition low when the measure is implemented and the re-entry questionnaire is sent.

In the add-on experiments, participants are selected from the core survey by the researchers, leading to a much more representative sample and increasing the external validity of the findings.

In analysing the effects of the top measures on levels of cycling, a repeated measures design is applied (with several before and after questionnaires). Variability in time is expected to be particularly high for distance and duration of cycling trips. We are confident that the target sample size and study design will allow us to detect significant changes in these variables as a result of the top measures because changes in duration of PA is the core input for computing health effects in each city.

## Ethics and dissemination

For each partner city the relevant permission to collect, store and process data was obtained from the local ethics committees. For the core module, the focus was on data handling and privacy aspects. For specific add-on modules, an amendment to the study design of the core module was approved by the involved local committees.

The protection of personal data is defined by national legislations and the European Directive 95/46/EC on the protection of individuals with regard to the processing of personal data and on the free movement of such data. The identity of participants is kept strictly confidential and stored securely in one place for all cities. Names are replaced by unique identifiers to anonymize the information. All participants have to agree explicitly with the conditions before registration. Data are strictly for scientific use.

Findings will be disseminated through scientific conferences and peer-reviewed journals, and more widely through newsletters, social media and the project website (www.pastaproject.eu) to participants, stakeholders and the general public. Popular media (audio-visual media, magazines, newspapers, and blogs) are also used for recruitment purposes.

## Conclusions

The Physical Activity through Sustainable Transport Approaches (PASTA) project aims to better understand AM, and to investigate how AM can reduce the health burden of physical inactivity. A longitudinal study design was chosen over a cross-sectional study to be able to investigate: (1) the correlates of AM; (2) the success factors for increasing AM and PA amongst a heterogeneous set of interventions; (3) if there is a substitution effect between PA from AM and PA from other domains, and if so, what the size of this effect is; and, (4) the health burden of traffic safety incidents associated with walking and cycling. In PASTA, add-on studies complement the longitudinal survey, collecting quantitative data on PA, travel behaviour, health status and traffic safety incidents. Innovative technologies such as an online data collection platform and smartphone applications are used for the collection of rich datasets that will be analysed using state of the art modelling techniques. This study therefore contributes to the advancement of cutting-edge technologies into future AM research, and to the improvement of future HIA on AM helping the decision making process in urban and transport planning.

### Ethics approval

Ethics approval was obtained for all aspects of the study by the local ethics committees in the countries where the work was conducted, and sent to the European Commission before the start of the survey/study.

The following committees approved the study:

- ▪Ethics board of the University Hospital of Antwerp (Belgium) on October 20, 2014
- ▪Clinical Research Ethics Committee of the Municipal Health Care (Barcelona – Spain) on October 1, 2014
- ▪Imperial College Research Ethics Committee (London – UK) on November 20, 2014
- ▪Regional ethical board, situated at the University of Lund (Oerebro – Sweden) on April 9, 2015
- ▪RSM - Roma Servizi per la Mobilità and the Air quality Commission of Roma Capitale Administration (Rome – Italy) on November 24, 2014
- ▪The Austrian Data Processing Register (Vienna – Austria) on September 26, 2014
- ▪Kantonale Ethikkommission Zürich (Switzerland) on October 28, 2014

## Additional files

Additional file 1:
**Supplemental material includes screenshots of the PASTA survey platform, a list of top measures selected in each city, and the design of the add-ons.** (PDF 1015 kb) Additional file 2:
**The full questionnaire of the PASTA longitudinal survey is provided as supplemental material, and is also available from** http://pastaproject.eu/fileadmin/editor-upload/sitecontent/City_survey/PASTA-questionnaires.pdf **.** (PDF 3792 kb)
