# Supplementary material for: Physical Activity through Sustainable Transport Approaches (PASTA): protocol for a multi-centre, longitudinal study
Source: BMC Public Health. 2015 Nov 14;15:1126. doi: 10.1186/s12889-015-2453-3 (PMC4650276; doi:10.1186/s12889-015-2453-3)
Supplement: Additional file 1: — Supplemental material includes screenshots of the PASTA survey platform, a list of top measures selected in each city, and the design of the add-ons. (PDF 1015 kb) [file 12889_2015_2453_MOESM1_ESM.pdf]

Supplemental material

Physical Activity through Sustainable Transport Approaches (PASTA): Protocol for a multi-centre, longitudinal study

Evi Dons[1,2], Thomas Götschi[3], Mark Nieuwenhuijsen[4,5,6], Audrey de Nazelle[7], Esther Anaya[7], Ione Avila-Palencia[4,5,6], Christian Brand[8], Tom Cole-Hunter[4,5,6], Mailin Gaupp-Berghausen[9], Sonja Kahlmeier[3], Michelle Laeremans[1,10], Natalie Mueller[4,5,6], Juan Pablo Orjuela[7], Elisabeth Raser[9], David Rojas-Rueda[4,5,6], Arnout Standaert[1], Erik Stigell[11], Tina Uhlmann[9], Regine Gerike[9,12], Luc Int Panis[1,10]

[1]Flemish Institute for Technological Research (VITO), Boeretang 200, 2400 Mol, Belgium

[2]Centre for Environmental Sciences, Hasselt University, Agoralaan building D, 3590 Diepenbeek, Belgium

[3]Physical Activity and Health Unit, Epidemiology, Biostatistics and Prevention Institute, University of Zurich, Seilergraben 49, 8001 Zurich, Switzerland

[4]Centre for Research in Environmental Epidemiology (CREAL), C/Dr. Aiguader 88, 08003 Barcelona, Spain

[5]Universitat Pompeu Fabra (UPF), C/Dr. Aiguader 88, 08003 Barcelona, Spain

[6]CIBER Epidemiología y Salud Pública (CIBERESP), C/Monforte de Lemos 3-5, 28029 Madrid, Spain

[7]Centre for Environmental Policy, Imperial College London, Exhibition Road, South Kensington Campus, SW7 2AZ London, United Kingdom

[8]University of Oxford (UOXF) – Transport Studies Unit, South Parks Road, Oxford, OX1 3QY, United Kingdom

[9]University of Natural Resources and Life Sciences Vienna, Institute for Transport Studies, Peter-Jordan-Straße 82, 1190 Vienna, Austria

[10]Transportation Research Institute (IMOB), Hasselt University, Wetenschapspark 5/6, 3590 Diepenbeek, Belgium

[11]Trivector Traffic AB, Stockholm, Sweden

[12]Dresden University of Technology, Chair of Integrated Transport Planning and Traffic Engineering, 01062 Dresden, Germany

Figure S 1: Screenshots of the registration process. (1) Choosing the city, (2) Registration

(1)

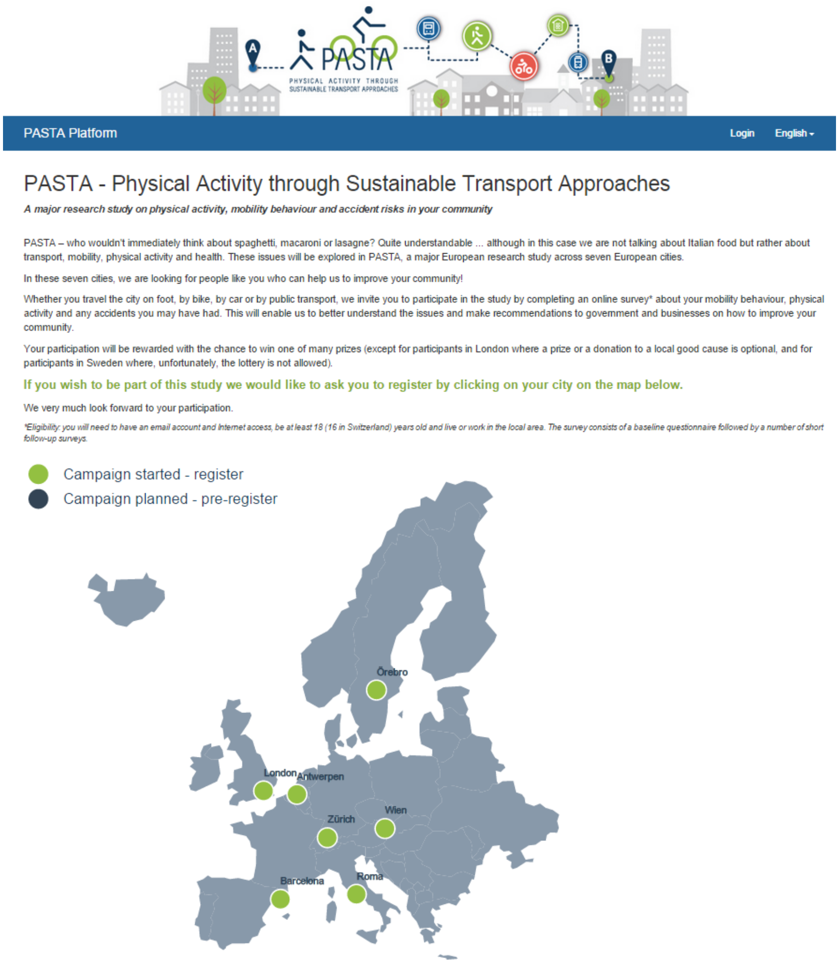

(2)

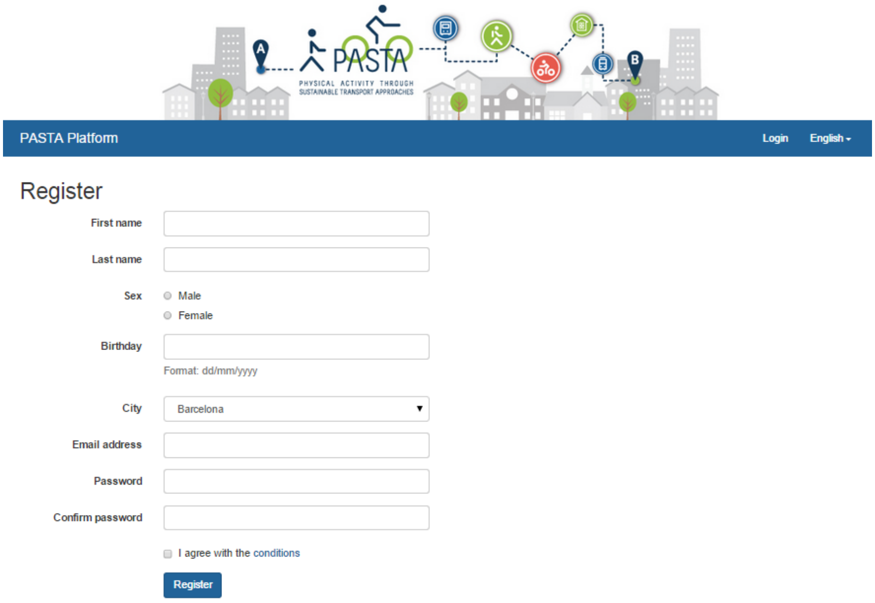

Figure S 2: Dashboard for participants with all the questionnaires that he/she has received

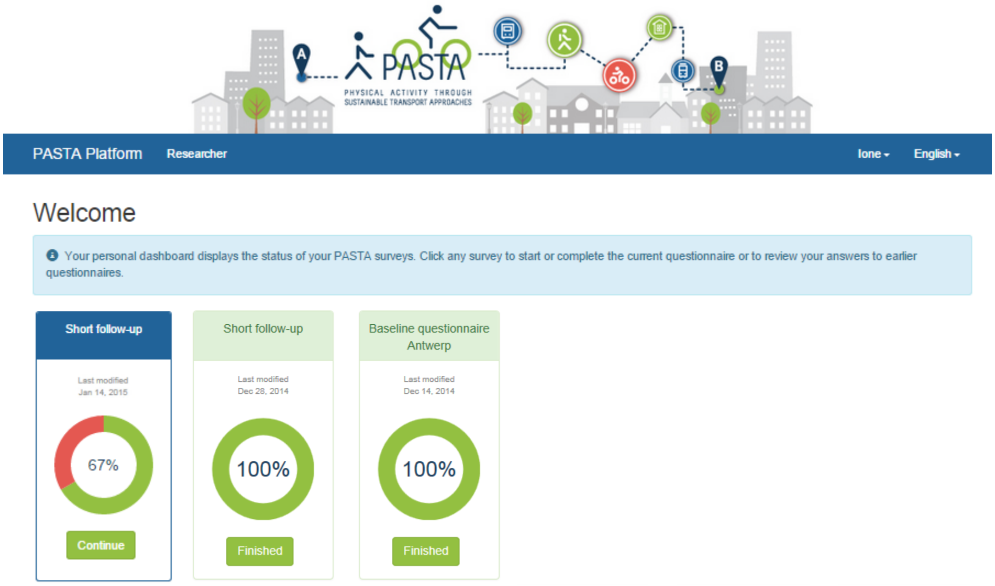

Figure S 3: Dashboard for researchers to monitor preregistration, registration and active participants (screenshot of situation on August 27, 2015)

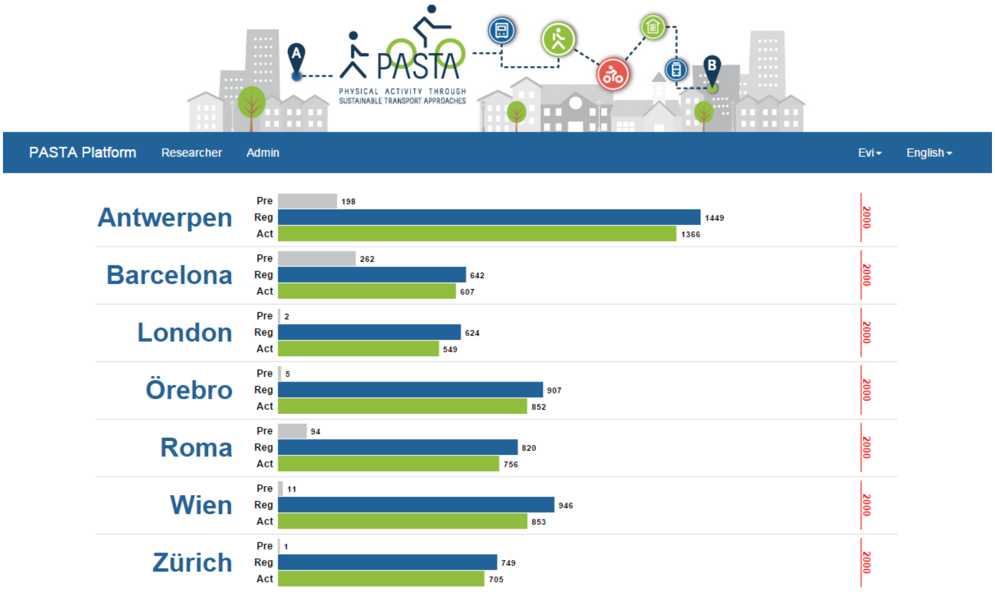

Figure S 4: Dashboard for researchers for a specific case study city (e.g. Barcelona), to monitor recruitment progress and sample characteristics (screenshot from May 20, 2015).

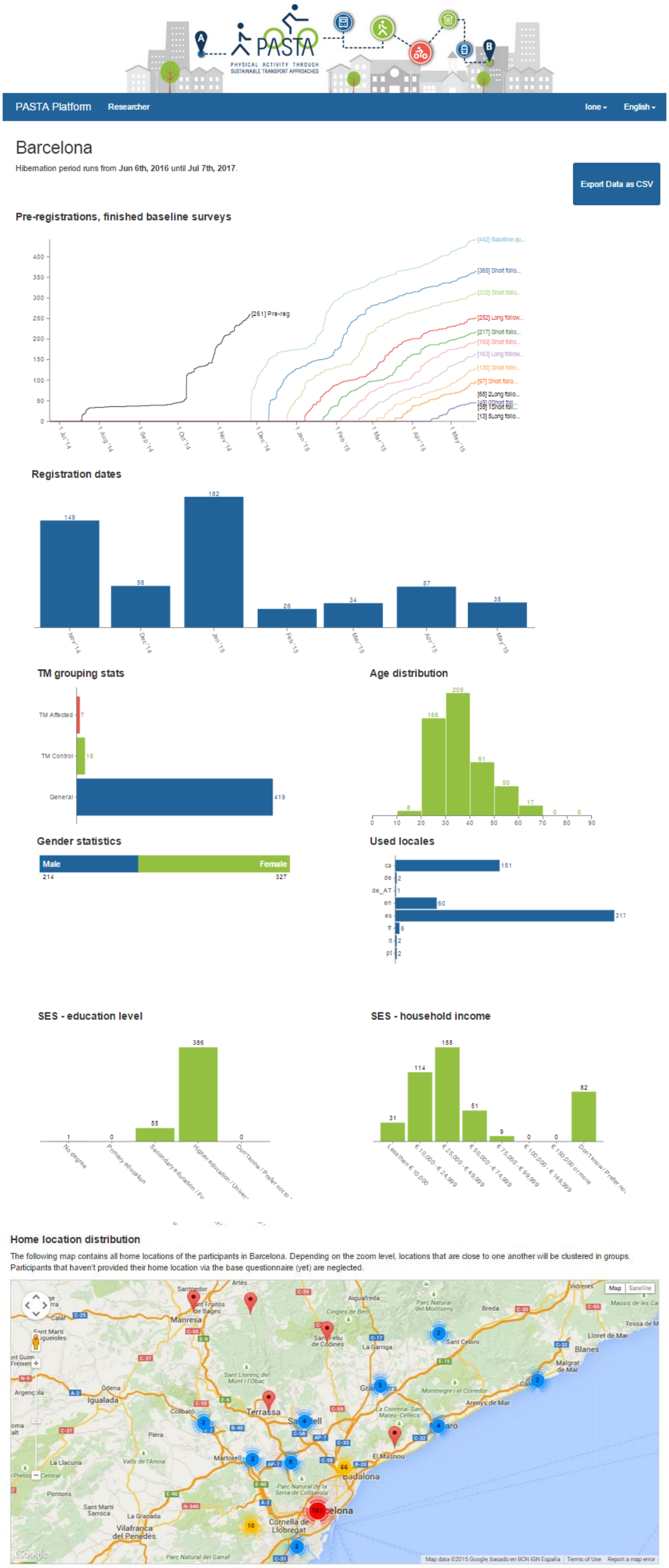

Table S 1: Selection of top measures to promote AT and PA studied in each PASTA city

City	Top measure
Vienna	Personalised mobility consultancy promoting walking and cycling
Zurich	E-bikes, Car sharing
Antwerp	Construction of a cycling bridge on a bicycle highway
Barcelona	Super-islands (Traffic-calmed residential areas)
Oerebro	Workplace mobility management
London	The redevelopment & opening of the London 2012 Olympic Park
Rome	Development of bicycle racks near high schools and public offices

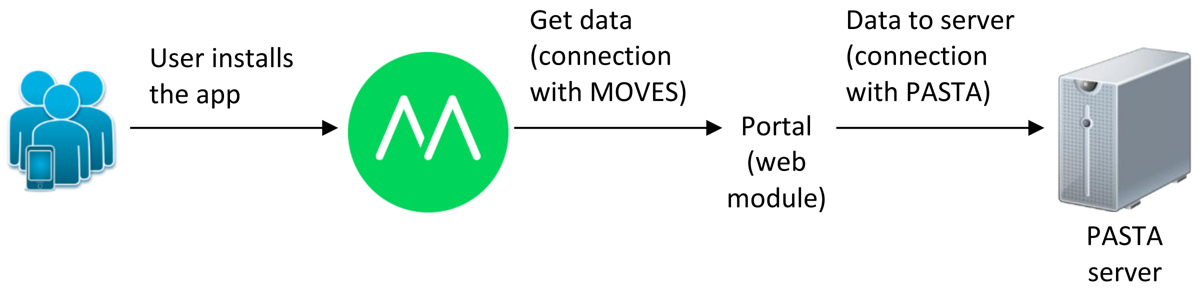

1. Accounts for PASTA participants are created on the MOVES website
2. User installs the MOVES app with the PASTA ID (email) and password
3. User collects data with the MOVES app
4. Through the portal, PASTA participants’ data is downloaded from the MOVES website
5. The portal forwards the data to the PASTA server

Figure S 5: Technical implementation of the route tracking app Moves within PASTA

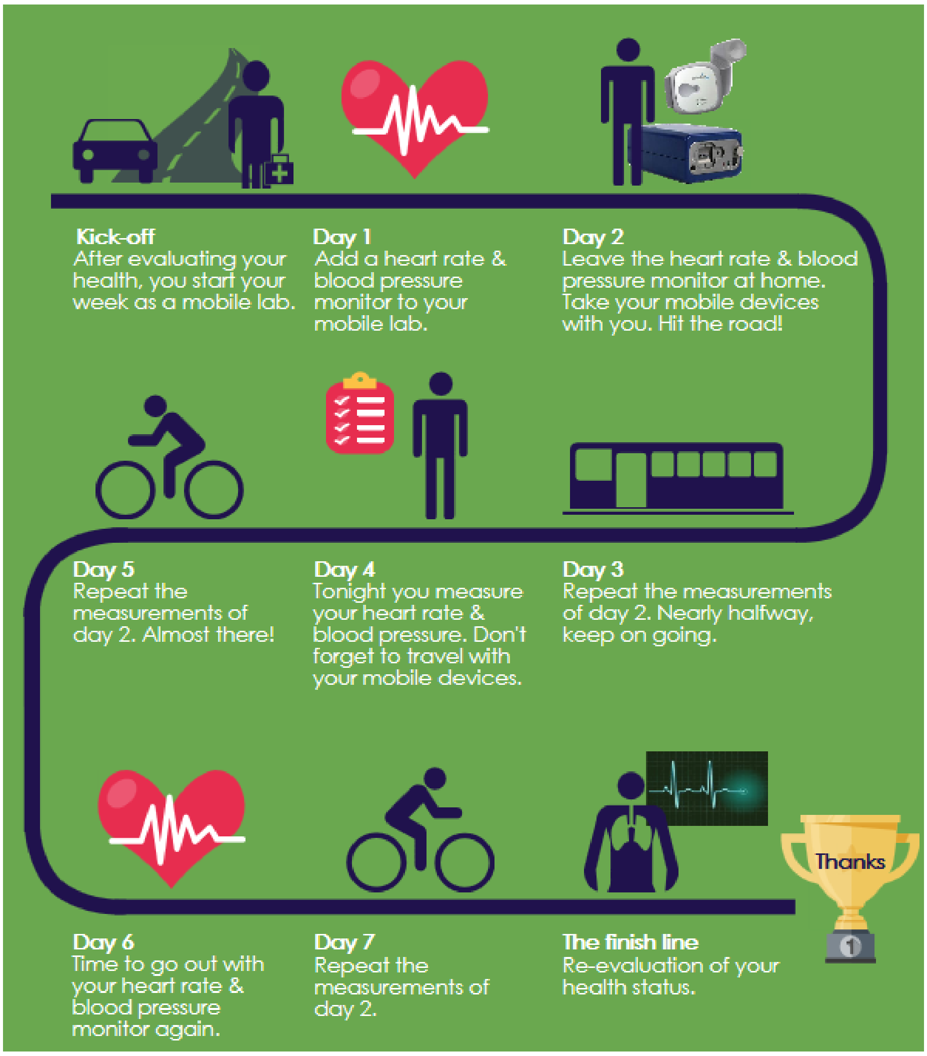

Figure S 6: Course of the measurement week for participants to the PASTA add-on module on PA, air pollution and short-term health effects
