# Supplementary material for: Physical Activity through Sustainable Transport Approaches (PASTA): protocol for a multi-centre, longitudinal study
Source: BMC Public Health. 2015 Nov 14;15:1126. doi: 10.1186/s12889-015-2453-3 (PMC4650276; doi:10.1186/s12889-015-2453-3)
Supplement: Additional file 2: — The full questionnaire of the PASTA longitudinal survey is provided as supplemental material, and is also available from http://pastaproject.eu/fileadmin/editor-upload/sitecontent/City_survey/PASTA-questionnaires.pdf . (PDF 3792 kb) [file 12889_2015_2453_MOESM2_ESM.pdf]

Overview of PASTA questionnaires

For more information on the PASTA project, visit http://www.pastaproject.eu/

Or contact one of the project partners:

- Coordinator: University of Natural Resources and Life Sciences (BOKU) - teampasta@boku.ac.at
- Coordinator of the longitudinal survey: Flemish Institute for Technological Research (VITO) – Prof dr Luc Int Panis – luc.intpanis@vito.be

This project has received funding from the European Union's Seventh Framework Programme for research, technological development and demonstration under grant agreement no 602624-2

Registration process

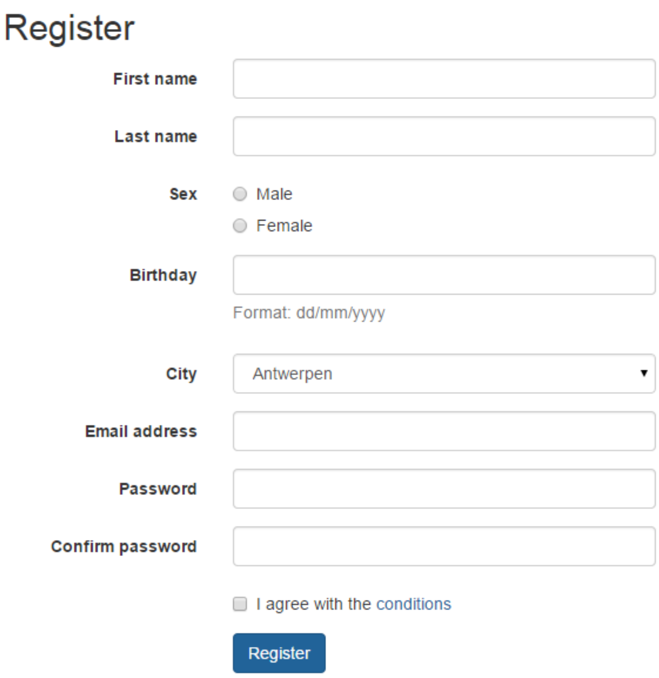

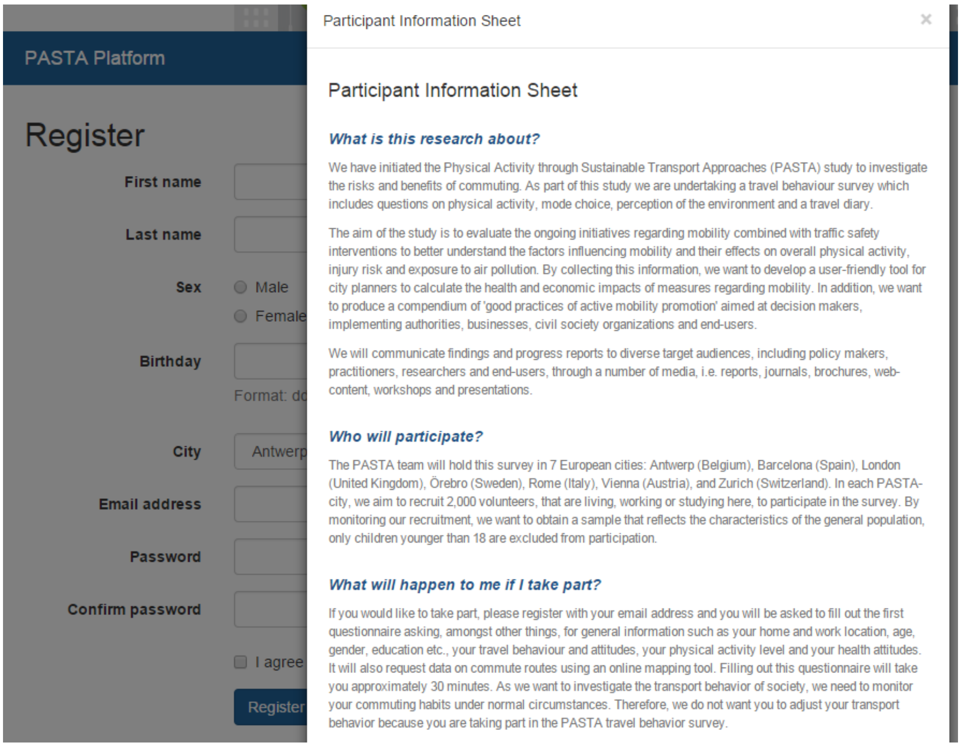

Baseline questionnaire

Baseline questionnaire Antwerp

2%

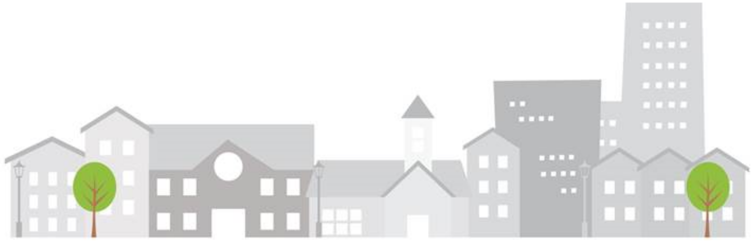

Welcome to the PASTA survey!

Completing this questionnaire will take about 20-30 minutes. For your convenience, you can leave the survey at any time, and continue at a later time. Once you have completed the survey you will be entered in a *prize draw.*

With many thanks and best wishes from the PASTA team.

Next ›

Baseline questionnaire Antwerp

5%

How did you find out about this survey?

- ○ Friends, neighbours or relatives
- ○ News or other media (newspaper, radio, TV, online news, etc.)
- ○ Employer/workplace
- ○ By mail (letter, mailout)
- ○ Social media (Facebook, Twitter, e-mail, online newsletter, etc.)
- ○ Local authority, government or other administration website (city, region, national, etc.)
- ○ Non-profit, non-governmental or other special interest organisation (website, e-mail, newsletter, handout, event)
- ○ Public notice at a store, the doctor, newsagent, etc.
- ○ I was contacted by telephone
- ○ I learned about the survey via outreach by the PASTA project (website, e-mail, event, T-shirts, etc.)
- ◉ Other

Please specify 'other'.

test

‹ Previous Next ›

Baseline questionnaire Antwerp

7%

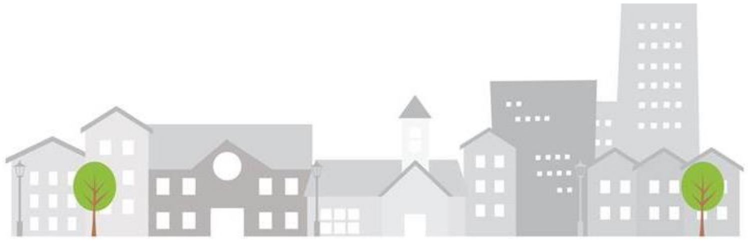

First, we would like to ask you some questions related to your mobility habits.

‹ Previous Next ›

Baseline questionnaire Antwerp

10%

Do you have a driver's licence for a car or van?

- ◉ Yes
- ○ No

Do you have access to a car or van? more info

- ◉ Always
- ○ Sometimes
- ○ Never

Are you a member of an official car sharing system or car club? more info

- ○ Yes
- ◉ No

Previous | Next

Baseline questionnaire Antwerp

12%

Have you used public transport at least once in the last year? more info

- ◉ Yes
- ○ No

What type of public transport ticket do you use most? more info

- ☐ One-way ticket, return ticket, day pass, short-trip ticket, multi-trip ticket, top-up card
- ☐ Weekly, monthly, semester, term or yearly pass
- ☐ Employment-related or student travel season ticket
- ☑ Other

From your home, how long would it take you to walk to the nearest public transport station or stop?

	Less than 1 minute	1 to 5 minutes	5 to 10 minutes	Approx. 15 minutes	Approx. 30 minutes	More than 30 minutes	Not applicable
Train	◉	○	○	○	○	○	○
Tram, Metro or Underground	◉	○	○	○	○	○	○
Bus or coach	◉	○	○	○	○	○	○

Previous | Next

Baseline questionnaire Antwerp

15%

Do you know how to ride a bicycle?

- ◉ Yes
- ○ No

Do you have access to a bicycle (private, or through a bike sharing system)?

- ◉ Yes
- ○ No

What type of bicycle do you use? more info

- ☐ City-bike, road bike, mountainbike
- ☐ Bike from bike sharing system
- ☐ Pedal-assisted electric bike (pedelec) for which motor cuts out at speeds over 25 kph (15 mph)
- ☐ Pedal-assisted electric bike (speed pedelec) for which motor continues to assist for speeds greater than 25 kph (15 mph) and up to 45 kph (27 mph)
- ☐ Other

Previous | Next

What type of bicycle do you use? more info

- ☐ City-bike, road bike, mountainbike
- ☐ Bike from bike sharing system
- ☑ Pedal-assisted electric bike (pedelec) for which motor cuts out at speeds over 25 kph (15 mph)
- ☐ Pedal-assisted electric bike (speed pedelec) for which motor continues to assist for speeds greater than 25 kph (15 mph) and up to 45 kph (27 mph)
- ☐ Other

Since when do you ride an electric bike?

- ◯ Less than 3 months
- ◯ Less than 1 year
- ◯ 1-3 years
- ◯ More than 3 years

What was your main motivation to start riding an electric bike?

- ◯ Less effort (than traditional cycling or walking)
- ◯ Faster (than traditional bike, public transport or car)
- ◯ Need or desire to travel longer distances
- ◯ Environmental considerations
- ◯ Health considerations
- ◯ Other

Thinking about the journeys that you use your electric bike now, what method of travel did you use *before* using an electric bike? more info

- ☐ Bicycle (not e-bike / pedelec)
- ☐ Motorcycle or moped
- ☐ Public transport
- ☐ Car or van
- ☐ None, I use my electric bike for recreational journeys I did not undertake before.
- ☐ Other

Compared to the methods of travel you used previously, how would you rate your sense of safety when riding your electric bike? (1 = much less safe; 10 = much safer)

0

Drag the marker to the right to indicate the exact number.

When riding your electric bike, do you usually wear a helmet?

- ◯ Yes
- ◯ No

Previous | Next

Baseline questionnaire Antwerp

17%

How often do you currently use each of the following methods of travel to get to and from places? more info

	Daily or almost daily	on 1-3 days per week	on 1-3 days per month	Less than once per month	Never	Don't know
Walk	◉	◯	◯	◯	◯	◯
Bicycle	◉	◯	◯	◯	◯	◯
Electric bicycle	◉	◯	◯	◯	◯	◯
Motorcycle or moped	◉	◯	◯	◯	◯	◯
Public transport	◉	◯	◯	◯	◯	◯
Car or van	◉	◯	◯	◯	◯	◯

Previous | Next

Baseline questionnaire Antwerp

20%

Listed below are potential activities that you may engage in in your daily life. Which method of travel would you be most likely to use?

	Walk	Bicycle	Motorcycle or moped	Public transport	Car or van	Other
Visiting friends or family in your city	◉	○	○	○	○	○
Shopping for groceries	◉	○	○	○	○	○
Going to a restaurant	◉	○	○	○	○	○
Taking a weekend excursion to a site/event in the city on a nice day	◉	○	○	○	○	○
Engaging in sports	◉	○	○	○	○	○

Previous   Next

Baseline questionnaire Antwerp

22%

For certain journeys that you take as part of your day-to-day travel you may have more than one method of travel available (e.g. car, bus, train, bicycle, walking). In general, how important are the following criteria for you when choosing a method of travel:

	Not important	Less important	Neutral	Important	Very important
Shorter travel time	◉	○	○	○	○
Lower travel cost	◉	○	○	○	○
Higher travel comfort	◉	○	○	○	○
Safer travel (with regards to traffic)	◉	○	○	○	○
Safer travel (with regards to crime)	◉	○	○	○	○
Lower exposure to air pollution	◉	○	○	○	○
Privacy	◉	○	○	○	○
Personal health benefits	◉	○	○	○	○
Low environmental impact	◉	○	○	○	○
Flexible departure time	◉	○	○	○	○
More predictable travel time and journey reliability	◉	○	○	○	○

Previous   Next

Baseline questionnaire Antwerp

25%

With your day-to-day travel needs in mind would you say that walking 'for travel' ...

	Very much disagree	Disagree	Neither agree nor disagree	Agree	Very much agree
It saves time.	◉	○	○	○	○
It is comfortable.	◉	○	○	○	○
is safe (with regards to traffic)	◉	○	○	○	○
It is safe (with regards to crime).	◉	○	○	○	○
It is unpleasant due to high levels of air pollution.	◉	○	○	○	○
It offers privacy.	◉	○	○	○	○
It offers personal health benefits.	◉	○	○	○	○
It offers flexibility (e.g. with regards to departure time).	◉	○	○	○	○
It offers a predictable travel time.	◉	○	○	○	○

Previous   Next

Baseline questionnaire Antwerp

27%

With your day-to-day travel needs in mind would you say that cycling 'for travel' ...

	**Very much disagree**	**Disagree**	**Neither agree nor disagree**	**Agree**	**Very much agree**
It saves time.	◉	○	○	○	○
It is comfortable.	◉	○	○	○	○
is safe (with regards to traffic)	◉	○	○	○	○
It is safe (with regards to crime).	◉	○	○	○	○
It is unpleasant due to high levels of air pollution.	◉	○	○	○	○
It offers privacy.	◉	○	○	○	○
It offers personal health benefits.	◉	○	○	○	○
It offers flexibility (e.g. with regards to departure time).	◉	○	○	○	○
It offers a predictable travel time.	◉	○	○	○	○

‹ Previous   Next ›

Baseline questionnaire Antwerp

30%

Do you agree with the following statements ...

	**Very much disagree**	**Disagree**	**Neither agree nor disagree**	**Agree**	**Very much agree**
I intend to walk more 'for travel' in the future.	◉	○	○	○	○
I intend to cycle more 'for travel' in the future.	◉	○	○	○	○
Over the last 12 months I have done more walking 'for travel' than in previous years.	◉	○	○	○	○
Over the last 12 months I have done more cycling 'for travel' than in previous years.	◉	○	○	○	○
In general, I try to walk for my day-to-day travel whenever possible.	◉	○	○	○	○
In general, I try to cycle for my day-to-day travel whenever possible.	◉	○	○	○	○

‹ Previous   Next ›

Baseline questionnaire Antwerp

32%

In a typical week, on how many days do you do a total of 30 min or more of physical activity, which is enough to raise your breathing rate?
This may include sport, exercise, and brisk walking or cycling for recreation or to get to and from places, but should **not** include housework or physical activity that may be part of your job.

2

‹ Previous   Next ›

Only for Zurich

Baseline questionnaire Zürich

35%

Imagine you are visiting a friend or family who lives in a building with an elevator. Assume that taking the elevator takes exactly the same time as taking the stairs.

Up to a maximum of how many floors would you take the stairs, instead of the elevator?

1

‹ Previous   Next ›

Baseline questionnaire Antwerp

35%

Where do you live? Please be as accurate as possible.

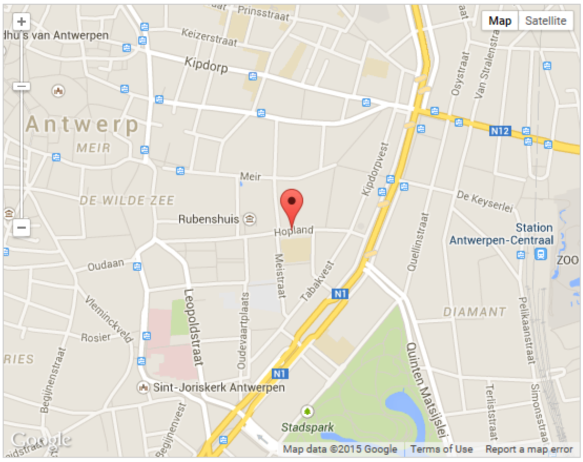

Click here to watch a video tutorial on how to use this form component.

You can drag the marker to a position on the map or you can use the search box below to pin a location. If your browser supports geolocation, you can also let it guess your location by clicking the ✥ button. Please keep in mind that only the position of the marker will be saved, not the address.

Search for a place or address

Hopland 26, 2000 Antwerpen, Belgium

❮ Previous Next ❯

Baseline questionnaire Antwerp

37%

What is your current employment status? more info

- ○ Full-time employed
- ◉ Part-time employed, or casual work
- ○ Student / In training
- ○ Home duties / Unemployed / Retired / Sickness leave / Parental leave
- ○ Don't know / Prefer not to answer

On average, approx. how many hours do you work per week?

What is the location of your main place of work? Please be as accurate as possible.

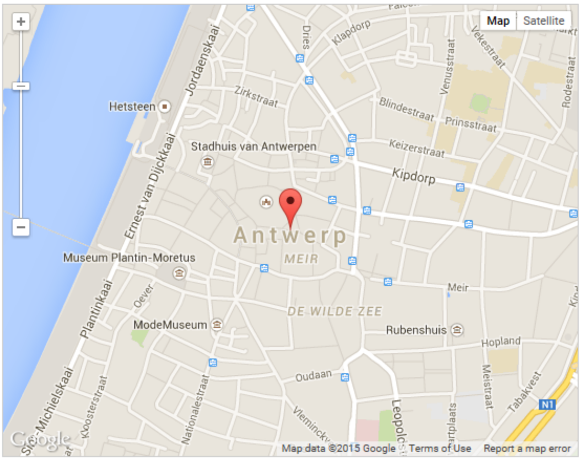

Click here to watch a video tutorial on how to use this form component.

You can drag the marker to a position on the map or you can use the search box below to pin a location. If your browser supports geolocation, you can also let it guess your location by clicking the ✥ button. Please keep in mind that only the position of the marker will be saved, not the address.

Search for a place or address

Eiermarkt 1-5, 2000 Antwerpen, Belgium

❮ Previous Next ❯

Baseline questionnaire Antwerp

40%

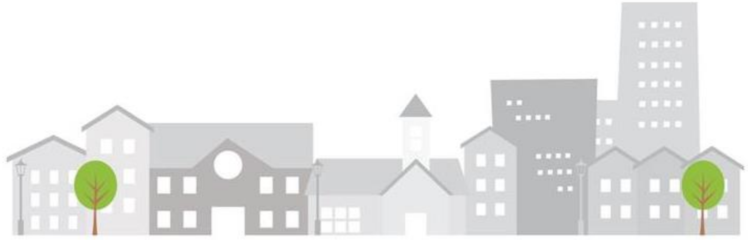

In the next section, please tell us about the journeys you did yesterday.

‹ Previous | Next ›

Baseline questionnaire Antwerp

42%

Travel diary

Please report all journeys you did **yesterday** in the order they occurred.

A journey is defined by a journey purpose (e.g. commuting, shopping, bringing kids to school, business travel, visiting friends or family). Once the purpose changes, a new journey begins. For each journey you may have used multiple methods of travel (for example, walking to the train station, taking the train, and walking to your final destination).

If you have no trips to report please click on 'next' to continue with the questionnaire.

Click here to watch a video tutorial on how to use this form component.

Click in the diary to add or edit a trip. Fill out the corresponding questions on start and end time, origin and destination, trip purpose, and method of transportation. When all questions on a trip are completed, click in the diary to add another trip. Details of a specific trip can be changed by selecting this trip in the diary. You can delete a single trip by selecting this trip in the diary, and clicking 'Remove trip'.

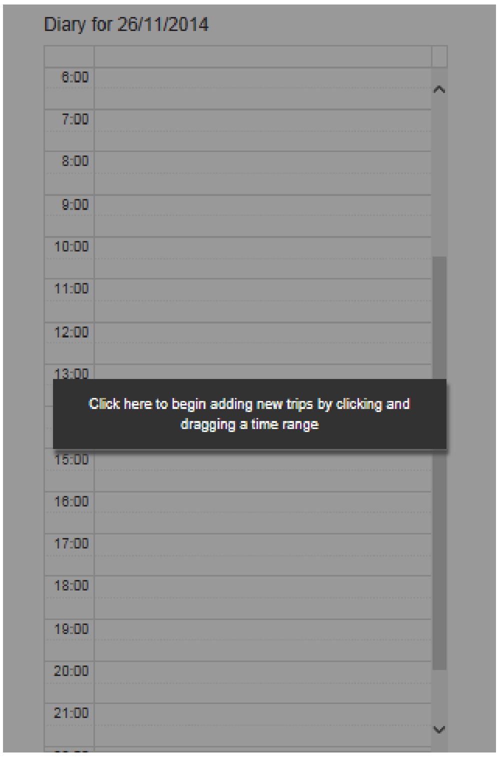

‹ Previous | Next ›

Remove journey

Start time 11 : 00 **End time** 11 : 25

Start time must be before end time and the journeys cannot overlap each other in time.

Start location

Search for a place or address more info

Hopland 26, 2000 Antwerpen, Belgium

Press enter or click on the magnifying glass after typing the address to update the location.

... or move the marker to a previously saved location

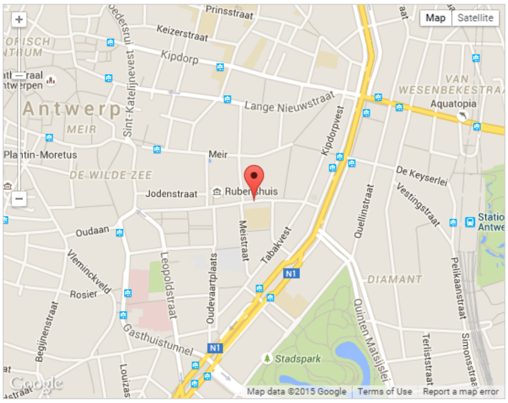

Journey Purpose

Return home

Your journey may be composed of different journey stages for which you used different methods of transport. Please insert all stages of your journey in the right chronological order.

Journey stage

Method of travel Bicycle

Duration (minutes) 25

Remove journey stage / leg

Add stage

End location

Search for a place or address more info

Nieuwpoortkaai 1, 2000 Antwerpen, Belgium

Press enter or click on the magnifying glass after typing the address to update the location.

... or move the marker to a previously saved location

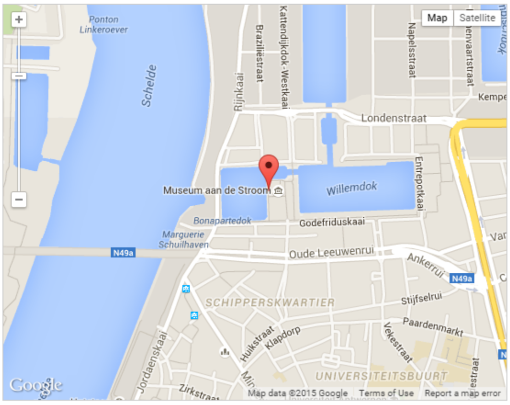

Save journey Remove journey

Baseline questionnaire Antwerp

45%

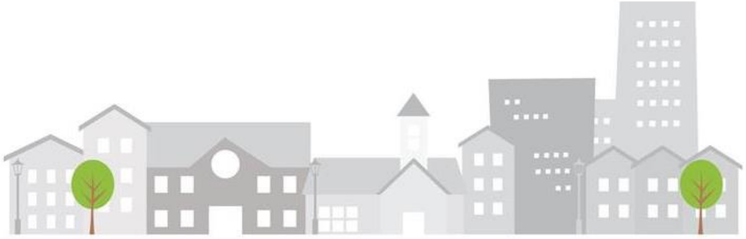

The questions in the next section of the survey are about how much physical activity you do in a typical week.

‹ Previous | Next ›

Baseline questionnaire Antwerp

47%

Activity at work

Think of work as the things that you have to do such as paid or unpaid work, study/training, and household chores or gardening.

Vigorous-intensity activities are activities that require hard physical effort and cause large increases in breathing or heart rate.

Moderate-intensity activities are activities that require moderate physical effort and cause small increases in breathing or heart rate.

Does your work involve vigorous-intensity activities for at least 10 minutes continuously? ℹ more info

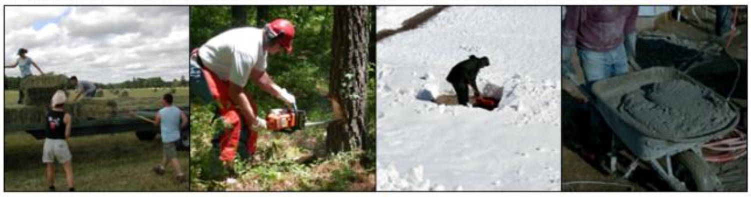

◉ Yes
○ No

In a typical week, on how many days do you do vigorous-intensity activities as part of your work?

Typically, how much time do you spend doing vigorous-intensity activities at work on such a day?

Please enter the duration as hours:minutes, separated by ":" (e.g., 2:30).

Does your work involve moderate-intensity activity for at least 10 minutes continuously? ℹ more info

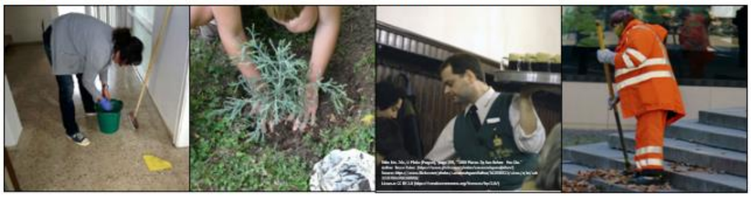

○ Yes
◉ No

‹ Previous | Next ›

Baseline questionnaire Antwerp

50%

Travel to and from places

The next questions exclude the physical activities at work that you have already mentioned.
Now think about the usual way you travel to and from places. Do *not* include walking for leisure, cycle tours or sports cycling.

Do you walk or use a bicycle for at least 10 minutes continuously to get to and from places?

- [x] Walk
- [ ] Bicycle
- [ ] Electric bicycle
- [x] No

In a typical week, on how many days do you walk for at least 10 minutes continuously to get to and from places?

Typically, how much time do you spend walking on such a day?

Please enter the duration as hours:minutes, separated by ":" (e.g., 2:30).

Previous | Next

Baseline questionnaire Antwerp

52%

Recreational activities

For the next questions exclude the work and transport activities that you have already mentioned. Now think about sports, fitness and recreational activities (leisure), including going for a walk or on a cycle tour.

Vigorous-intensity activities are activities that require hard physical effort and cause large increases in breathing or heart rate.

Moderate-intensity activities are activities that require moderate physical effort and cause small increases in breathing or heart rate.

Do you do any vigorous-intensity sports, fitness or recreational (leisure) activities for at least 10 minutes continuously? more info

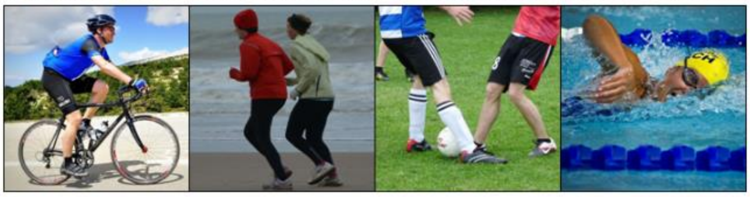

- (•) Yes
- ( ) No

In a typical week, on how many days do you do vigorous-intensity sports, fitness or recreational (leisure) activities?

Typically, how much time do you spend doing vigorous-intensity sports, fitness or recreational activities on such a day?

Please enter the duration as hours:minutes, separated by ":" (e.g., 2:30).

Do you do any moderate-intensity sports, fitness or recreational (leisure) activities for at least 10 minutes continuously? more info

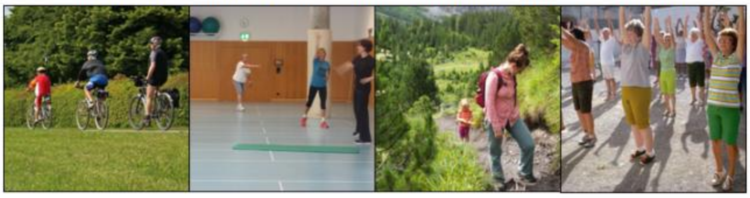

- ( ) Yes
- (•) No

Previous | Next

Baseline questionnaire Antwerp

55%

Sedentary behaviour

The following question is about sitting or reclining at work, at home, getting to and from places, or with friends. Time spent sleeping should not be included.

For example: time spent sitting at a desk; eating; travelling in car, bus or train; reading; watching television; or using the computer.

How much time do you usually spend sitting or reclining on a typical day?

10:00

Please enter the duration as hours:minutes, separated by ":" (e.g., 2:30).

‹ Previous | Next ›

Baseline questionnaire Antwerp

57%

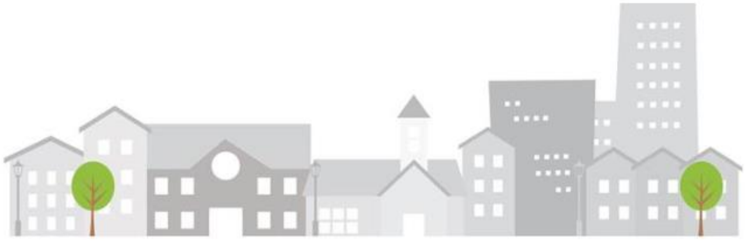

We now have some questions about you and your health.

‹ Previous | Next ›

Baseline questionnaire Antwerp

60%

How tall are you?

cm

Use a point or comma as decimal separator

How much do you weigh?

kg

Use a point or comma as decimal separator

In general, how would you say your health is?

- (●) Excellent
- ( ) Very good
- ( ) Good
- ( ) Fair
- ( ) Poor
- ( ) Don't know / Prefer not to answer

‹ Previous | Next ›

Baseline questionnaire Antwerp

62%

Do you smoke? more info

- ( ) Yes
- ( ) No, but I used to smoke
- (●) No, I have never smoked

How many glasses of alcohol do you drink in a typical week? more info

0

Drag the marker to the right to indicate the exact number.

‹ Previous | Next ›

Baseline questionnaire Antwerp

65%

In the past week, how often have you suffered from:

	Never	Sometimes	Regularly	Often	Very often	Don't know / Prefer not to answer
Dizziness/light-headedness	◉	○	○	○	○	○
Back and/or shoulder pain	◉	○	○	○	○	○
Headache	◉	○	○	○	○	○
Painful muscles	◉	○	○	○	○	○
Chest pain	◉	○	○	○	○	○
Nausea	◉	○	○	○	○	○
Pain in stomach or abdomen	◉	○	○	○	○	○
Fatigue	◉	○	○	○	○	○

Previous Next

Baseline questionnaire Antwerp

67%

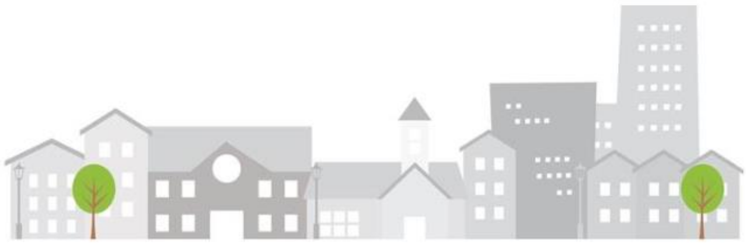

Next, we would like to learn about some of your attitudes, beliefs and opinions.

Previous Next

Baseline questionnaire Antwerp

70%

Are you worried that air pollution in the neighbourhood of either your home or work can lead to health problems?

- ○ Not worried at all
- ○ Not worried
- ○ Neither worried nor not worried
- ◉ Worried
- ○ Extremely worried

How much are you disturbed, bothered or annoyed by noise from road traffic in the neighbourhood of either your home or work?

- ◉ Not annoyed at all
- ○ Not annoyed
- ○ Neither annoyed nor not annoyed
- ○ Annoyed
- ○ Extremely annoyed

Previous Next

Baseline questionnaire Antwerp

72%

Do you agree with the following statements …

	Very much disagree	Disagree	Neither agree nor disagree	Agree	Very much agree
Most people who are important to me think that I should walk 'for travel'.	◉	○	○	○	○
Most people who are important to me think that I should cycle 'for travel' (that is, getting from place to place).	◉	○	○	○	○
I feel morally responsible to walk in order to decrease the negative effects on the environment that motorized methods of travel have.	◉	○	○	○	○
I feel morally responsible to use a bicycle in order to decrease the negative effects on the environment that motorized methods of travel have.	◉	○	○	○	○
In my neighbourhood walking is well regarded.	◉	○	○	○	○
In my neighbourhood cycling is well regarded.	◉	○	○	○	○

‹ Previous | Next ›

Baseline questionnaire Antwerp

75%

Do you agree with the following statements …

	Very much disagree	Disagree	Neither agree nor disagree	Agree	Very much agree
For me, walking would be difficult in everyday life.	◉	○	○	○	○
For me, using a bicycle would be difficult in everyday life.	◉	○	○	○	○
Walking 'for travel' is something I do automatically without really thinking about it.	◉	○	○	○	○
Cycling 'for travel' is something I do automatically without really thinking about it.	◉	○	○	○	○
I am fit enough to walk.	◉	○	○	○	○
I am fit enough to cycle.	◉	○	○	○	○

‹ Previous | Next ›

Baseline questionnaire Antwerp

77%

Do you agree with the following statements …

	Very much disagree	Disagree	Neither agree nor disagree	Agree	Very much agree
Personal circumstances make it impossible for me to walk more (e.g. family or work commitments, carrying luggage, escorting children).	◉	○	○	○	○
Personal circumstances make it impossible for me to cycle more (e.g. family or work commitments, carrying luggage, escorting children).	◉	○	○	○	○
Inadequate parking for my bike at home and at my destinations make it impossible for me to cycle more.	◉	○	○	○	○
The lack of changing and shower facilities at my destinations prevents me from using a bicycle.	◉	○	○	○	○
The organisation of my everyday life requires me to travel a lot.	◉	○	○	○	○
I have to travel all the time to meet my obligations.	◉	○	○	○	○

‹ Previous | Next ›

Baseline questionnaire Antwerp

80%

Do you agree with the following statements ...

	Very much disagree	Disagree	Neither agree nor disagree	Agree	Very much agree
Regardless of what other people do, my own values and principles oblige me to walk 'for travel' whenever possible.	◉	○	○	○	○
Regardless of what other people do, my own values and principles oblige me to cycle 'for travel' whenever possible.	◉	○	○	○	○
In my neighbourhood it is common for people to walk 'for travel'.	◉	○	○	○	○
In my neighbourhood it is common for people to cycle 'for travel'.	◉	○	○	○	○

❮ Previous | Next ❯

Baseline questionnaire Antwerp

82%

Rate the following statements ...

	Very weak	Weak	Neutral	Strong	Very strong
My intention to walk 'for travel' is ...	◉	○	○	○	○
My intention to cycle 'for travel' is ...	◉	○	○	○	○

❮ Previous | Next ❯

Baseline questionnaire Antwerp

85%

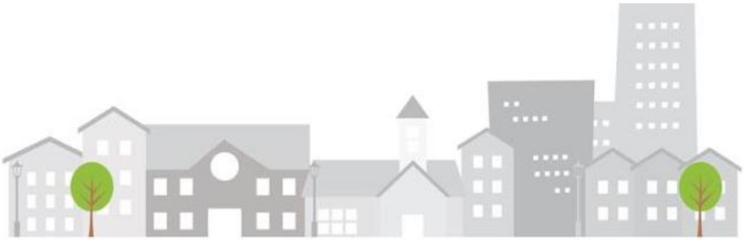

Before we finish, we have a few more questions about you and your household.

❮ Previous | Next ❯

Baseline questionnaire Antwerp

87%

How many people live in your household? Please include yourself, and fill in the exact number for each.

How many children under 6 years of age

0

Drag the marker to the right to indicate the exact number.

How many children aged 6-17 years

0

Drag the marker to the right to indicate the exact number.

How many adults aged 18-65 years

2

Drag the marker to the right to indicate the exact number.

How many adults aged over 65 years

0

Drag the marker to the right to indicate the exact number.

Previous | Next

Only for London:

Baseline questionnaire London

87%

Please tick one box to describe your ethnic background.

- ○ Asian
- ○ Black/African/Caribbean
- ○ White
- ○ Mixed/multiple ethnic groups
- ○ Arab
- ○ Latin-American
- ○ Other
- ○ Don't know / Prefer not to answer

Previous | Next

Baseline questionnaire Antwerp

90%

What nationality are you?

Belgium

What was your father's nationality when you were born?

Belgium

What was your mother's nationality when you were born?

Belgium

Previous | Next

Baseline questionnaire Antwerp

92%

What is your highest level of completed education?

- ○ No degree
- ○ Primary education
- ◉ Secondary education / Further education
- ○ Higher education / University education
- ○ Don't know / Prefer not to answer

What was your total household income after taxes during the past 12 months? more info

- ○ Less than € 10,000
- ○ € 10,000 - € 24,999
- ◉ € 25,000 - € 49,999
- ○ € 50,000 - € 74,999
- ○ € 75,000 - € 99,999
- ○ € 100,000 - € 149,999
- ○ € 150,000 or more
- ○ Don't know / Prefer not to answer

Previous | Next

Only for London:

Baseline questionnaire London

95%

We have one last page with questions specifically for residents living in London.

Have you recently received any kind of information or material which encourages people to walk and cycle?

- ○ Yes
- ◉ No

Are you aware of any new walking and cycling schemes, facilities or other improvement schemes for walking or cycling in your community?

- ○ Yes
- ◉ No

Do you use any new walking and/or cycling routes or other new schemes which have improved walking and/or cycling in your community?

- ○ Yes
- ◉ No

All the participants in the PASTA project in London (that is, those who have completed at least one questionnaire) are invited to participate in the Celebratory Event at the end of the study (2017). In addition, if you fill in more than three questionnaires, your participation can be recognise with any of the following options, please choose one:

- ◉ I would like to participate in the prize draw which will be drawn every 3 months and will consist of vouchers from general stores.
- ○ I would like my participation to be rewarded by giving a donation to a local wellbeing charity, at the end of the project.
- ○ I do not wish to receive a reward nor do I wish to give a donation for my participation, I am happy to contribute to the study and help improve my community.

Would you like to be informed about future events and opportunities to participate in PASTA project studies? (please note: your details will not be passed to a third party or be used for any other purpose) more info

- ◉ Yes
- ○ No

Previous | Next

Only for Oerebro:

Baseline questionnaire Örebro

95%

We have one more question specifically for inhabitants of (the wider region of) Örebro.

Part of the this study will be specifically addressed at employees at certain workplaces. Do you currently work at any of these workplaces?

- (•) Region Örebro län: Regionservice Eklundavägen (Eklundavägen 1, 2 eller 11, Örebro)
- ( ) Region Örebro län: Regionservice på USÖ (Södra Grev Rosengatan, Örebro)
- ( ) Region Örebro län: avdelningarna: Special kemi, öppna akut-lab, Hornhinnebanken på USÖ (Södra Grev Rosengatan, Örebro)
- ( ) Region Örebro län: avdelningarna: Strategisk samordning, Utveckling och säkerhet, Sterilcentralen, Arbets- och miljömedicin, Specialkemi, Kardiologiska kliniken, Reumatologiska kliniken på USÖ (Södra Grev Rosengatan, Örebro)
- ( ) Axfood (Handelsgatan 5, Örebro)
- ( ) Örebro Kommun: Stadsbyggnadshusen (Åbylundsgatan 8 A-B eller Tomtagatan 9, Örebro)
- ( ) Örebro Kommun: Socialförvaltningen (Ribbingsgatan 1-3, Örebro)
- ( ) None of these workplaces

‹ Previous | Next ›

Only for Vienna:

Baseline questionnaire Vienna

95%

We have one more question specifically for inhabitants of (the wider region of) Vienna.

In the past 12 months, have any of the following life changing events happened to you? more info

- [x] Moved house
- [x] I received medical advice to increase my physical activity
- [ ] Other life changing event or events
- [ ] No life changing event

In what month did you move? (1 = January; 12 = December)

In which month did you get the medical advice? (1 = January; 12 = December)

Are you interested in learning more about the positive impacts of physical activity? We can show you opportunities for integrating physical activity into your daily routines. This will help you to live longer, look better and with a better quality of life.

- ( ) Yes
- ( ) No

‹ Previous | Next ›

Only for Antwerp:

Baseline questionnaire Antwerp

95%

We have one last page with questions specifically for inhabitants of (the wider region of) Antwerp.

The province of Antwerp has built, and is still extending, the so called '**fietsostrades**' or cycling highways network. These supra-local high quality cycle ways run next to railways, canals, etc. Currently there are 15 cycling highways in the province of Antwerp. The cycling paths are designed to provide fast, direct, safe and comfortable travel.

Had you heard of the 'fietsostrades' before completing this survey?

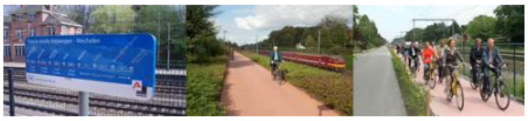

- ◉ Yes
- ○ No

How often do you use them?

- ○ Daily or almost daily
- ○ on 1-3 days per week
- ○ on 1-3 days per month
- ○ Less than once per month
- ◉ Never or almost never
- ○ Don't know

‹ Previous | Next ›

How often do you use them?

- ○ Daily or almost daily
- ○ on 1-3 days per week
- ◉ on 1-3 days per month
- ○ Less than once per month
- ○ Never or almost never
- ○ Don't know

Think about your last trip on the fietsostrade. Include the trip even if you used the fietsostrade only for a short distance. What 'fietsostrade' did you use?

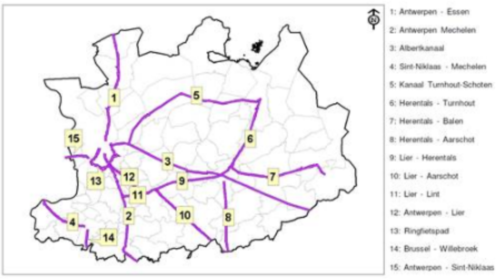

- ○ Fietsostrade Antwerpen-Essen (nr 1)
- ○ Fietsostrade Antwerpen-Mechelen (nr 2)
- ○ Ringfietspad (nr 13)
- ○ Other 'fietsostrade'

What was the approx. distance of the complete trip (one way)? ℹ more info

[ ] km

Use a point or comma as decimal separator

How long did it take you (one way, in minutes)? ℹ more info

[ ]

What was the purpose of this journey?

- ○ Recreation, health or fitness
- ○ Commuting / travel to or from work
- ○ Travel to or from education (school, higher or further education)
- ○ Travel in the course of business
- ○ Shopping or personal business (e.g. doctor, bank, solicitor and post office)
- ○ Visiting friends and relatives or travel for other social activities

‹ Previous | Next ›

Baseline questionnaire Antwerp

97%

Thank you for completing this questionnaire!

You are now enrolled in our lottery; we will contact you through email if you are among the lucky ones winning a prize!

In two weeks time you will be invited by email to complete a very short follow-up survey, which will take less than 5 minutes.

As part of the PASTA project we also conduct research using new innovative measurement devices. These devices study your mobility, your physical activity, or measure health indicators or air pollution. Would you be interested in participating in one of these studies? Read more... more info

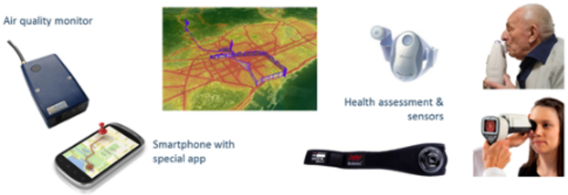

○ Yes
○ No

Are you interested in the results of the project?

○ Yes, I want to receive a summary of the most important results. This will be sent to me by the end of the study.
○ No

Previous | Finish

Follow-up questionnaire - short

Short follow-up

14%

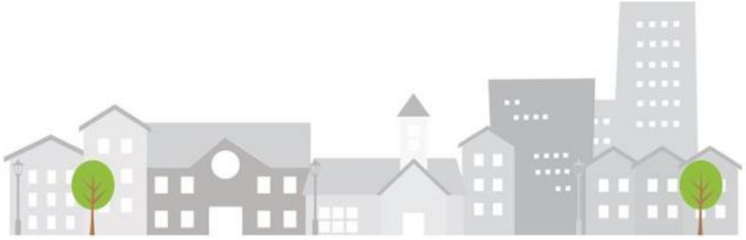

Today we would like to ask you a short set of key questions regarding your travel and physical activities. This questionnaire will take only about 5 minutes to complete.

Next

Short follow-up

28%

In the last 7 days, on how many days did you use each of the following methods of travel to get to and from places? more info

	Did not use it	on 1-3 days per week	on 4-5 days per week	on 6-7 days per week
Walk	◉	○	○	○
Bicycle	◉	○	○	○
Electric bicycle	◉	○	○	○
Motorcycle or moped	◉	○	○	○
Public transport	◉	○	○	○
Car or van	◉	○	○	○

Previous Next

Short follow-up

42%

In the last 7 days, on how many days have you done a total of 30 min or more of physical activity that was enough to raise your breathing rate?
This may include sport, exercise, and brisk walking or cycling for recreation or to get to and from places by walking or cycling, but should **not** include housework or physical activity that may be part of your job.

4

Previous Next

Short follow-up

57%

Now we would like to ask in more detail about your walking and cycling journeys in the last 7 days. Think about the usual way you travel to and from places. Please do **not** include walking for recreational or leisure purposes, bike tours or cycling for sports.

In the last 7 days, did you walk or use a bicycle for at least 10 minutes continuously to get to and from places?

- [x] Walk
- [ ] Bicycle
- [ ] Electric bicycle
- [ ] No

In the last 7 days, on how many days did you walk for at least 10 minutes continuously to get to and from places?

Typically, how much time do you spend walking on such a day?

Please enter the duration as hours:minutes, separated by ":" (e.g., 2:30).

Previous | Next

Only in Follow-up 1:

Short follow-up

71%

Think about the location you visited most often in the last 7 days. Can you indicate the route you took to reach this destination as accurately as possible? more info

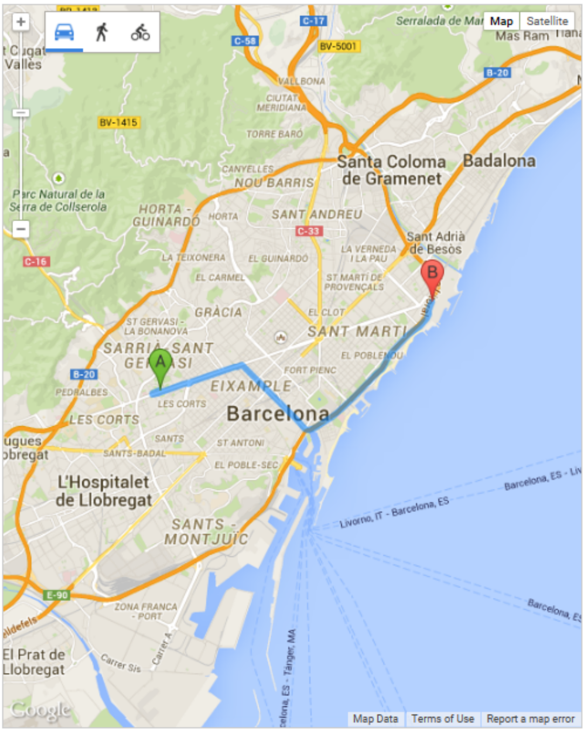

Click here to watch a video tutorial on how to use this form component.

You can drag the markers to a position on the map or you can use the search boxes below to set the starting point and destination. If your browser supports geolocation, you can also let it guess your location by clicking the ✥ buttons. It is possible to adjust a proposed route by dragging it. Please keep in mind that only the positions of the markers will be saved, not the addresses.

A - Starting point

Search for a place or address

Avinguda Diagonal, 644, 08017 Barcelona, Barcelona, Spain

... or move the marker to a previously saved location

B - Destination

Search for a place or address

Passeig Marítim de la Mar Bella, 08019 Barcelona, Barcelona, Spa

... or move the marker to a previously saved location

I travelled this route by...

- ( ) Walk
- (•) Bicycle
- ( ) Electric bicycle
- ( ) Motorcycle or moped
- ( ) Public transport
- ( ) Car or van
- ( ) Other

Previous | Next

Short follow-up

85%

Since the last time you filled out a questionnaire for the PASTA study, have you experienced any safety relevant incidents (i.e. a collision, fall, or near miss as a pedestrian, cyclist, in public transport, or driving)? Note that we are interested in all incidents, whatever the purpose of your journey.

- (•) Yes
- ( ) No

We would appreciate if you could report some details regarding any such incident(s). Please select the type(s) of incident(s) below by clicking the 'Add' button; additional questions will then open automatically. The incident questionnaire takes about 10 minutes to complete (per incident).

As a pedestrian...

A collision (i.e. you were hit by a vehicle incl. bicycles) or a fall (i.e. you hit the ground without involvement of others).

Add

A near miss (i.e. an unexpected event while walking which forced you or another party to take sudden evasive action, without which a collision would have occurred).

Add

As a cyclist (incl. electric bikes)...

A collision (i.e. you were hit by a vehicle, or you hit a vehicle or a pedestrian) or a fall (i.e. you hit the ground, or you collided with something without involvement of other persons).

Add

A near miss (i.e. an unexpected event while cycling which forced you or another party to take sudden evasive action, without which a collision would have occurred).

Add

As a passenger on a public transport vehicle (bus, metro, tram)...

A collision by the vehicle (i.e. the vehicle you were in hit or was hit by another vehicle, bicycle or pedestrian).

Add

A fall inside the vehicle (i.e. you fell inside the vehicle because of a sudden stop or similar).

Add

As a driver or passenger of a car/van...

A collision without personal injury (i.e. you were hit by a vehicle, or you hit an obstacle, a vehicle or a pedestrian, but you did not get injured).

Add

A collision with personal injury (i.e. you were hit by a vehicle, or you hit an obstacle, a vehicle or a pedestrian, and you got injured, requiring at least some treatment).

Add

Previous | Finish ✔

Follow-up questionnaire - long

Long follow-up

11%

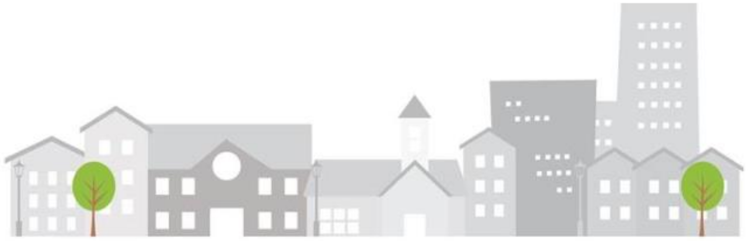

Today we would like to ask you a few key questions regarding your recent travel and physical activities. This questionnaire will take about 15 minutes to complete.

Next

Long follow-up

22%

In the last 7 days, on how many days did you use each of the following methods of travel to get to and from places? more info

	Did not use it	on 1-3 days per week	on 4-5 days per week	on 6-7 days per week
Walk	○	◉	○	○
Bicycle	○	◉	○	○
Electric bicycle	○	○	◉	○
Motorcycle or moped	○	○	○	◉
Public transport	○	○	◉	○
Car or van	○	○	◉	○

Previous Next

Long follow-up

33%

Activity at work

Think of work as the things that you have to do such as paid or unpaid work, study/training, and household chores or gardening.

Vigorous-intensity activities are activities that require hard physical effort and cause large increases in breathing or heart rate.

Moderate-intensity activities are activities that require moderate physical effort and cause small increases in breathing or heart rate.

Does your work involve vigorous-intensity activities for at least 10 minutes continuously? more info

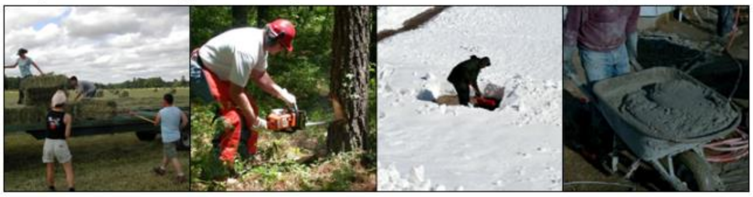

- (●) Yes
- ( ) No

In the last 7 days, on how many days did you do vigorous-intensity activities as part of your work?

0

Typically, how much time do you spend doing vigorous-intensity activities at work on such a day?

Please enter the duration as hours:minutes, separated by ":" (e.g., 2:30).

Does your work involve moderate-intensity activity for at least 10 minutes continuously? more info

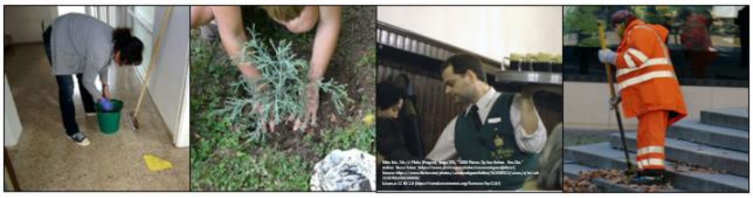

- ( ) Yes
- (●) No

‹ Previous | Next ›

Long follow-up

44%

Travel to and from places

The next questions exclude the physical activities at work that you have already mentioned.
Now think about the usual way you travel to and from places. Do *not* include walking for leisure, cycle tours or sports cycling.

In the last 7 days, did you walk or use a bicycle for at least 10 minutes continuously to get to and from places?

- [ ] Walk
- [x] Bicycle
- [ ] Electric bicycle
- [ ] No

In the last 7 days, on how many days did you use a cycle for at least 10 minutes continuously to get to and from places?

Typically, how much time do you spend cycling on such a day?

Please enter the duration as hours:minutes, separated by ":" (e.g., 2:30).

‹ Previous | Next ›

Long follow-up

55%

Recreational activities

For the next questions exclude the work and transport activities that you have already mentioned. Now think about sports, fitness and recreational activities (leisure), including going for a walk or on a cycle tour.

Vigorous-intensity activities are activities that require hard physical effort and cause large increases in breathing or heart rate.

Moderate-intensity activities are activities that require moderate physical effort and cause small increases in breathing or heart rate.

Do you do any vigorous-intensity sports, fitness or recreational (leisure) activities for at least 10 minutes continuously? more info

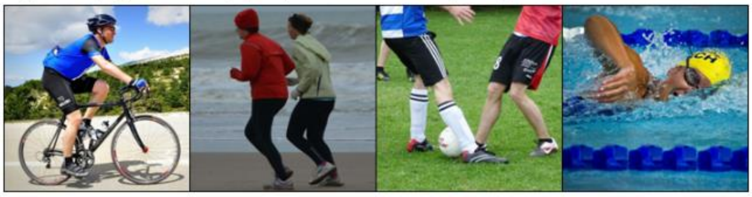

(•) Yes
( ) No

In the last 7 days, on how many days did you do vigorous-intensity sports, fitness or recreational (leisure) activities?

Typically, how much time do you spend doing vigorous-intensity sports, fitness or recreational activities on such a day?

Please enter the duration as hours:minutes, separated by ":" (e.g., 2:30).

Do you do any moderate-intensity sports, fitness or recreational (leisure) activities for at least 10 minutes continuously? more info

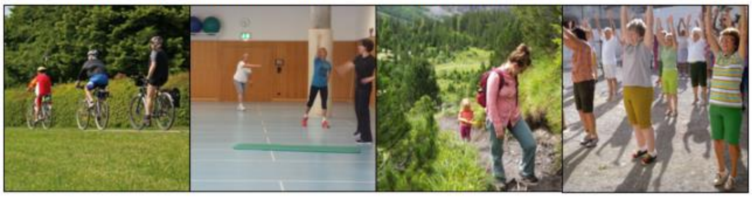

( ) Yes
(•) No

Previous | Next

Long follow-up

66%

Sedentary behaviour

The following question is about sitting or reclining at work, at home, getting to and from places, or with friends. Time spent sleeping should not be included.

For example: time spent sitting at a desk; eating; travelling in car, bus or train; reading; watching television; or using the computer.

In the last 7 days, how much time did you spend sitting or reclining on a typical day?

0:20

Please enter the duration as hours:minutes, separated by ":" (e.g., 2:30).

Previous | Next

Travel diary

Please report all journeys you did **yesterday** in the order they occurred.

A journey is defined by a journey purpose (e.g. commuting, shopping, bringing kids to school, business travel, visiting friends or family). Once the purpose changes, a new journey begins. For each journey you may have used multiple methods of travel (for example, walking to the train station, taking the train, and walking to your final destination).

If you have no trips to report please click on 'next' to continue with the questionnaire.

Click here to watch a video tutorial on how to use this form component.

Click in the diary to add or edit a trip. Fill out the corresponding questions on start and end time, origin and destination, trip purpose, and method of transportation. When all questions on a trip are completed, click in the diary to add another trip. Details of a specific trip can be changed by selecting this trip in the diary. You can delete a single trip by selecting this trip in the diary, and clicking 'Remove trip'.

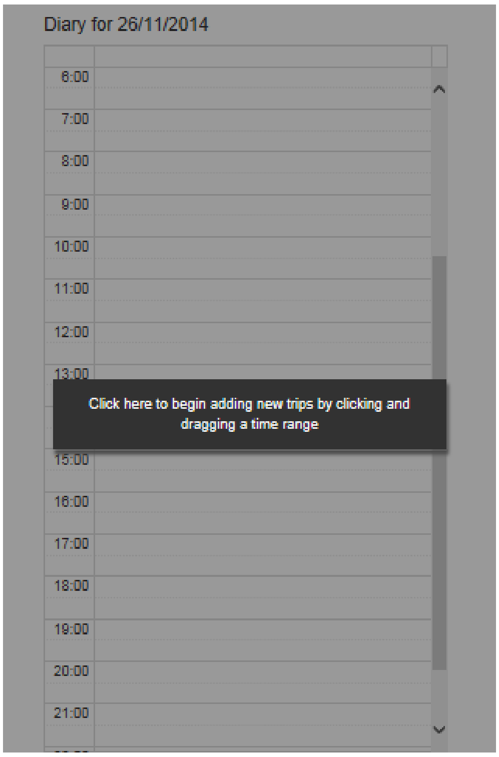

Previous

Next

Remove journey

Start time 11 : 00 **End time** 11 : 25

Start time must be before end time and the journeys cannot overlap each other in time.

Start location

Search for a place or address more info

Hopland 26, 2000 Antwerpen, Belgium

Press enter or click on the magnifying glass after typing the address to update the location.

... or move the marker to a previously saved location

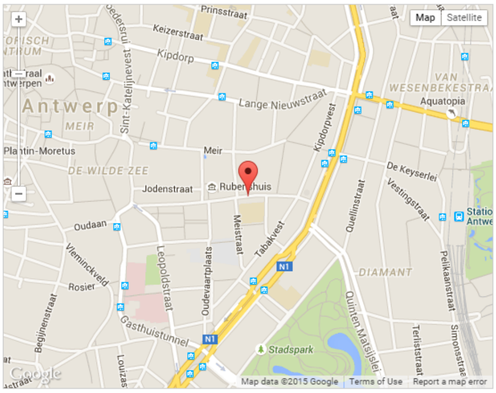

Journey Purpose

Return home

Your journey may be composed of different journey stages for which you used different methods of transport. Please insert all stages of your journey in the right chronological order.

Journey stage

**Method of travel**	Bicycle
**Duration (minutes)**	25

Remove journey stage / leg

Add stage

End location

Search for a place or address more info

Nieuwpoortkaai 1, 2000 Antwerpen, Belgium

Press enter or click on the magnifying glass after typing the address to update the location.

... or move the marker to a previously saved location

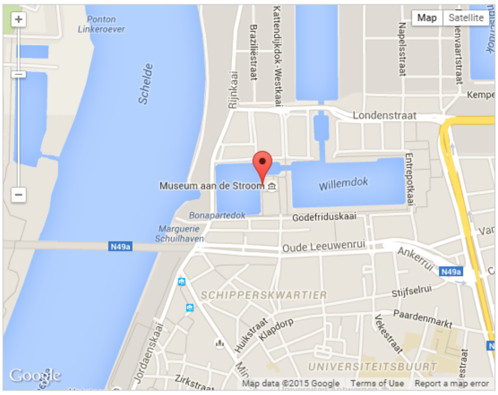

Save journey Remove journey

Long follow-up

88%

Since the last time you filled out a questionnaire for the PASTA study, have you experienced any safety relevant incidents (i.e. a collision, fall, or near miss as a pedestrian, cyclist, in public transport, or driving)? Note that we are interested in all incidents, whatever the purpose of your journey.

- (•) Yes
- ( ) No

We would appreciate if you could report some details regarding any such incident(s). Please select the type(s) of incident(s) below by clicking the 'Add' button; additional questions will then open automatically. The incident questionnaire takes about 10 minutes to complete (per incident).

As a pedestrian...

A collision (i.e. you were hit by a vehicle incl. bicycles) or a fall (i.e. you hit the ground without involvement of others).

Add

A near miss (i.e. an unexpected event while walking which forced you or another party to take sudden evasive action, without which a collision would have occurred).

Add

As a cyclist (incl. electric bikes)...

A collision (i.e. you were hit by a vehicle, or you hit a vehicle or a pedestrian) or a fall (i.e. you hit the ground, or you collided with something without involvement of other persons).

Add

A near miss (i.e. an unexpected event while cycling which forced you or another party to take sudden evasive action, without which a collision would have occurred).

Add

As a passenger on a public transport vehicle (bus, metro, tram)...

A collision by the vehicle (i.e. the vehicle you were in hit or was hit by another vehicle, bicycle or pedestrian).

Add

A fall inside the vehicle (i.e. you fell inside the vehicle because of a sudden stop or similar).

Add

As a driver or passenger of a car/van...

A collision without personal injury (i.e. you were hit by a vehicle, or you hit an obstacle, a vehicle or a pedestrian, but you did not get injured).

Add

A collision with personal injury (i.e. you were hit by a vehicle, or you hit an obstacle, a vehicle or a pedestrian, and you got injured, requiring at least some treatment).

Add

Previous | Finish

Pedestrian & bike crash questionnaire

6%

We would like to ask you to provide some details about the bicycle crash. This information will help us to investigate crash risks for cycling, and to identify effective measures to avoid them. Answering these questions will take you about 10 minutes.

When did the crash occur (approximately)?

14/01/2015 13:25:13

Use the format DD/MM/YYYY HH:mm:ss e.g. 31/12/2014 12:59:59

What was the main purpose of the journey when the bike crash occurred ?

- ○ Return home
- ○ To work (commuting)
- ○ For business / in the course of work
- ○ To school or place of study
- ◉ Shopping
- ○ Personal business/running errands (e.g. going to a post office, hospital, doctor's surgery or bank)
- ○ Pick up/drop off/accompanying or escorting others
- ○ Social/leisure (e.g. visiting friends or family, going to a fitness club, tennis court, cinema or public park - Note this excludes recreational travel)
- ○ Cycling for recreation, fitness or health
- ○ Cycling for sport
- ○ Other

With regards to traffic safety, how did you feel the minute before the crash happened? (0 = very unsafe; 10 = very safe)

7

Drag the marker to the right to indicate the exact number.

back to questionnaire

Next

12%

Have you ever used the route on which the crash occurred before?

- ○ No, never
- ○ Yes, once
- ◉ Yes, several times
- ○ Yes, quite often

Please indicate the route you cycled to the crash location (you do not need to report the use of other modes of transport). more info

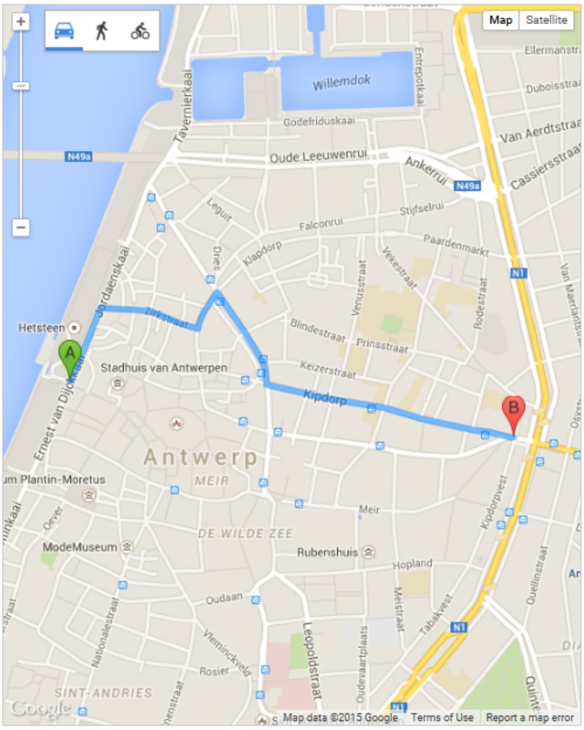

Click here to watch a video tutorial on how to use this form component.

You can drag the markers to a position on the map or you can use the search boxes below to set the starting point and destination. If your browser supports geolocation, you can also let it guess your location by clicking the ✥ buttons. It is possible to adjust a proposed route by dragging it. Please keep in mind that only the positions of the markers will be saved, not the addresses.

A - Starting point

Search for a place or address

Ernest van Dijckkaai 10, 2000 Antwerpen, Belgium

... or move the marker to a previously saved location

B - Destination

Search for a place or address

Sint-Jacobsmarkt 97, 2000 Antwerpen, Belgium

... or move the marker to a previously saved location

Does the final route on the map match the route you took?

- ○ Yes
- ◉ Yes, approximately
- ○ No
- ○ I don't remember the exact route

If the route on the map is inaccurate, could you please describe the route you took in your own words using streetnames and indicating turns you took. more info

back to questionnaire | Previous | Next

18%

The next questions refer to the circumstances of the crash.

How would you describe the location of the crash?

Along a street

- ☐ It was along a street.
- ☐ It was along a one-way street, but opposite the allowed direction of travel.
- ☑ It was when crossing the street (not at an intersection).

At an intersection

- ☐ It was at a crossing without traffic lights.
- ☐ It was at an intersection with traffic lights.
- ☐ It was at a roundabout.

At another location

- ☐ It was at a bus/tram/metro/train stop/station.
- ☐ It was in a pedestrian zone / shared zone with mixed traffic / residential street.
- ☐ It was at a car park.
- ☐ It was along (or when crossing) a driveway.
- ☐ It was off the street (beside the street, sidewalk, bicycle path without connection to the street network).
- ☐ Other
- ☐ Don't know

back to questionnaire | Previous | Next

25%

What types of bicycle infrastructure are there at this location?

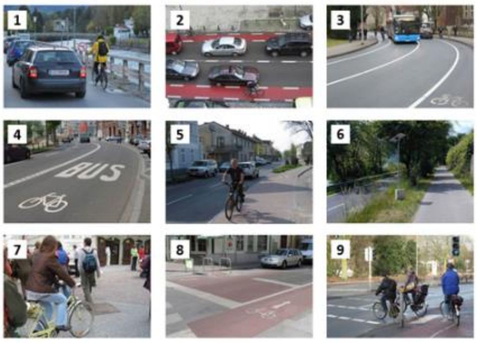

- [ ] 1 - No bicycle Infrastructure: No facilities for cyclists.
- [ ] 2 - Bike lane - dashed line: Bicycle lane is marked with a dashed line.
- [ ] 3 - Bike lane - solid line: Bicycle lane is marked with solid lines on the street surface.
- [ ] 4 - Shared lane: Markings on the street surface indicate space shared by bicycle and bus or taxi.
- [ ] 5 - Cycle track: Parallel path for cyclists alongside streets. Separated by a physical barrier.
- [x] 6 - Separate path: a segregated path which can be used by cyclists and pedestrians.
- [ ] 7 - Mixed use street, path or space, where cyclists and pedestrians (with or without cars) share the same space.
- [ ] 8 - Intersection markings: Ground markings specifically for cyclists.
- [ ] 9 - Intersection signals and signs: Signals and/or signs specifically for cyclists.
- [ ] Other

Were you using this bicycle infrastructure?

- ○ Yes
- ◉ No

Why didn't you use the dedicated infrastructure for cyclists?

- ○ I was crossing the street.
- ○ It was too narrow.
- ○ I felt unsafe there.
- ○ It wasn't available for the direction I was going to (e.g. at an intersection).
- ○ It would have been a time-consuming detour for me.
- ○ It would have forced me to move slower than I wanted to.
- ○ The quality of the surface was poor.
- ◉ It was blocked (e.g. by parked vehicles, snow or road works).
- ○ The width was fair.
- ○ Other
- ○ Don't know

Were you using this bicycle infrastructure?

- ◉ Yes
- ○ No

How did you perceive the width of it close to the crash location?

- ○ It was too narrow.
- ○ The width was fair.
- ○ It was wider than necessary.
- ○ Don't know

back to questionnaire | Previous | Next

31%

Was someone else involved in the crash?

- [ ] Nobody. I fell, lost balance, collided with an obstacle, or similar.
- [x] Yes, a pedestrian was involved.
- [ ] Yes, a cyclist was involved.
- [ ] Yes, a vehicle was involved (car, public transport, etc.).

Did the person use roller skates / skateboard / segway or similar?

- ○ Yes
- ○ No

If more than 2 parties were involved, please specify them. more info

back to questionnaire | Previous | Next

37%

Was one of the reasons for the crash the behavior of another person?

( ) Yes
(•) No

Was one of the reasons for the crash the infrastructure or the condition of the infrastructure?

( ) Yes
(•) No

Was one of the reasons for the crash your own behaviour / conditions of your own bike?

( ) Yes
(•) No

Please briefly describe in your own words how the crash happened. more info

back to questionnaire | Previous | Next

Was one of the reasons for the crash the behavior of another person?

(•) Yes
( ) No

Why do you think the crash happened? more info

- [ ] Behaviour of other drivers, cyclists or road users.
- [ ] Behaviour of pedestrians.
- [ ] Other vehicles / drivers / cyclists disobeyed a traffic law.
- [ ] A pedestrian disobeyed a traffic law.

Was one of the reasons for the crash the infrastructure or the condition of the infrastructure?

(•) Yes
( ) No

Why do you think the crash happened? more info

- [ ] Slipping / sliding (on a wet, icy, snowy, sandy etc. surface).
- [x] Unevenness in the street surface (e.g. pothole).
- [ ] Unevenness or obstacle along my direction of travel (e.g. kerb, rail, cobblestones).
- [x] Collision with an obstacle (e.g. tree, bollard, traffic sign, post, wall).
- [ ] Restricted view of traffic conditions (e.g. due to parked vehicles).
- [ ] Traffic routing / street design.

With what did you collide?

( ) A tree
( ) A bollard
( ) Traffic sign, post, pillar, pole, etc.
( ) A wall
( ) A crash barrier, guide post
( ) Other
( ) Don't know

What type of unevenness was it? more info

- [ ] Roadworks
- [ ] Tracks (tram / train)
- [ ] A kerb
- [ ] A pothole
- [ ] A manhole cover
- [ ] Edge of the street
- [ ] Other
- [ ] Don't know

Was one of the reasons for the crash your own behaviour / conditions of your own bike?

(•) Yes
( ) No

Why do you think the crash happened? more info

- [ ] My own behaviour (e.g. cycled too fast, misjudged the situation, braked too strongly).
- [ ] I was not attentive (unobservant, lost in thought, distracted, in a hurry, sleepy etc.).
- [ ] I was inattentive because I was talking on my phone, listening to music using a headphone or earbuds, or looking at my smartphone.
- [ ] I disobeyed a traffic law (e.g. I ignored a red traffic light).
- [ ] Problems with luggage / loading on the bicycle.
- [ ] Defect on the bicycle.
- [ ] Other
- [ ] Don't know

43%

Were you under the influence of alcohol at the time of the crash?

- ○ No
- ◉ A little
- ○ Moderately
- ○ Seriously
- ○ Don't know / Prefer not to answer

Were you travelling alone or accompanied by others?

- ○ I was travelling alone.
- ◉ I was travelling in someone's company.

How many persons did accompany / escort you? more info

Did you have to attend one of the accompanying persons? (e.g. of children or disabled persons)

- ◉ Yes
- ○ No

back to questionnaire | Previous | Next

Only for bike crashes:

50%

What best describes the type of bike you were using at the time of the crash?

- ○ City bike
- ○ Mountain bike
- ◉ Racing bike
- ○ Pedal-assisted electric bike (pedelec) for which motor cuts out at speeds over 25 kph (15 mph)
- ○ Pedal-assisted electric bike (speed pedelec) for which motor continues to assist for speeds greater than 25 kph (15 mph) and up to 45 kph (27 mph)
- ○ Bike from bike sharing system
- ○ Other

Which of the following gear did you wear at the time of the crash? more info

- ☑ Helmet
- ☐ Bike lights (front and/or rear)
- ☐ Reflective clothing, or clothing with reflective stripes
- ☐ Other
- ☐ None of these options
- ☐ Don't know

In general, how would you rate your overall cycling skills? (0 = very bad; 10 = very good)

8

Drag the marker to the right to indicate the exact number.

back to questionnaire | Previous | Next

56%

Please describe the light conditions at the time of the crash.

- ○ Night, no lighting
- ○ Night, with lighting
- ◉ Dawn, dusk
- ○ Day (light)
- ○ Other
- ○ Don't know

Please describe the weather at the time of the crash. more info

- ☑ Dry
- ☐ Light rain / rain
- ☐ Heavy rain / hail
- ☐ Snow
- ☐ Fog (visibility less than 100m)
- ☐ Strong wind
- ☐ Bright daylight
- ☐ Other
- ☐ Don't know

Please describe the surface material at the time / location of the crash. more info

- ☑ Concrete, asphalt
- ☐ Gravel, sand
- ☐ Cobblestones
- ☐ Paving stone
- ☐ Loose paving
- ☐ Other
- ☐ Don't know

Please describe the surface conditions at the time / location of the crash. more info

- ☑ Dry
- ☐ Damp
- ☐ Wet, with puddles
- ☐ Ice or snow
- ☐ Dirt (leafs, mud, sand, gravel, etc.)
- ☐ Other
- ☐ Don't know

back to questionnaire | Previous | Next

62%

Please describe the motorized traffic conditions at the time and location of the crash.

- ○ There was lots of vehicle traffic (crossing the street would have been possible only with long waiting times).
- ○ There was some vehicle traffic (crossing the street would have been easy).
- ◉ Vehicle traffic was low.
- ○ No vehicles at all.
- ○ Don't know

What were the cyclist traffic conditions at the time and place of the crash?

- ◉ There were a lot of cyclists.
- ○ There were some cyclists.
- ○ Cyclist traffic was low.
- ○ No cyclists at all.
- ○ Don't know

What were the pedestrian traffic conditions at the time and place of the crash?

- ○ There was lots of pedestrian traffic.
- ○ There was some pedestrian traffic.
- ○ Pedestrian traffic was virtually non-existent.
- ◉ No pedestrians at all.
- ○ Don't know

If you wish, you can make your own suggestion on how the crash could have been prevented.

back to questionnaire | Previous | Next

68%

The following questions refer to the consequences of the crash.

Did you suffer from a physical injury as a result of the crash? (a bruise / a cramp is also considered to be an injury)

- (•) Yes
- ( ) No

Physical injuries

Select body parts by checking them in the list or by clicking on the images.

- ☐ Head
- ☐ Neck
- ☐ Upper right arm
- ☐ Right forearm
- ☐ Right hand or fingers
- ☐ Upper left arm
- ☐ Left forearm
- ☐ Left hand or fingers
- ☐ Belly / abdomen / perineum
- ☐ Upper right leg
- ☐ Lower right leg
- ☐ Right foot or toes
- ☐ Upper left leg
- ☐ Lower left leg
- ☐ Left foot or toes
- ☐ Lumbar region
- ☐ Left buttock or pelvis
- ☐ Face
- ☐ Right shoulder
- ☐ Right elbow
- ☐ Right wrist
- ☐ Left shoulder
- ☐ Left elbow
- ☐ Left wrist
- ☐ Thorax
- ☐ Right hip or groin
- ☐ Right knee
- ☐ Right ankle or heel
- ☐ Left hip or groin
- ☐ Left knee
- ☐ Left ankle or heel
- ☐ Back
- ☐ Right buttock or pelvis

Front

Back

Type of physical damage: more info

- ☐ Graze(s)
- ☐ Fracture(s)
- ☐ Bruise(s)
- ☐ Sprain(s)
- ☐ Muscular injury(ies)
- ☐ Cut(s)
- ☐ Burn(s)
- ☐ Concussion
- ☐ Lost consciousness
- ☐ Shock
- ☐ Other

back to questionnaire | Previous | Next

75%

Did you receive medical treatment after the crash?

- ( ) No
- ( ) Yes, I treated it myself or by another person (no doctor).
- ( ) Yes, I went to a doctor or hospital myself.
- ( ) Yes, from an ambulance at the location of the crash.
- ( ) Yes, I was brought to the hospital for medical treatment but could go home the same day.
- (•) Yes, I was hospitalized (min. 1 night in the hospital).

How many nights were you hospitalised?

Were you incapable of working / going to school for at least one day just after you had your crash?

- (•) Yes
- ( ) No

For how many days you have been incapable of working / going to school?

back to questionnaire | Previous | Next

81%

Is there an official police report about the crash?

- ( ) Yes, the police showed up and they officially reported the crash.
- ( ) Yes, I reported the crash later to the police (in the station, by phone or online).
- (•) No, the police showed up but they didn't officially report the crash.
- ( ) No, the police didn't show up and the crash was not officially reported.
- ( ) Don't know

Has the crash been reported to an insurance company? more info

- ( ) Yes
- (•) No
- ( ) Don't know

back to questionnaire | Previous | Next

87%

Did or do you intend to adapt your behaviour in one of the following ways because of the crash? more info

- [ ] I now take a different route.
- [ ] I now use a different method of travel.
- [ ] I travel at other times, e.g. not at night.
- [ ] I pay more attention.
- [ ] I ride more slowly.
- [ ] I improve my bicycle (e.g. repair lights).
- [ ] I adapt my outfit (e.g. wear a helmet or reflective clothing).
- [ ] Other
- [x] No

back to questionnaire | Previous | Next

93%

Thank you very much for all the information on the crash you had! This will help us to analyse crash risks and to develop measures to reduce them.
For the future we wish you pleasant and safe journeys!

Please click on 'Finish' and you will return to the main questionnaire where you have the choice between reporting any further incidents or completing the questionnaire.

Cancel incident | Previous | Finish
